# Macroscopic and high-throughput printing of aligned nanostructured polymer semiconductors for MHz large-area electronics

**DOI:** 10.1038/ncomms9394

**Published:** 2015-09-25

**Authors:** Sadir G. Bucella, Alessandro Luzio, Eliot Gann, Lars Thomsen, Christopher R. McNeill, Giuseppina Pace, Andrea Perinot, Zhihua Chen, Antonio Facchetti, Mario Caironi

**Affiliations:** 1 Center for Nano Science and Technology @PoliMi, Istituto Italiano di Tecnologia, Via Pascoli 70/3, Milano 20133, Italy; 2 Politecnico di Milano, Dipartimento di Fisica, P.za L. da Vinci 32, Milano 20133, Italy; 3 Department of Materials Science and Engineering, Monash University, Wellington Road, Clayton, Victoria 3800, Australia; 4 Australian Synchrotron, 800 Blackburn Road, Clayton, Victoria 3168, Australia; 5 Polyera Corporation, 8045 Lamon Avenue, Skokie, Illinois 60077, USA

## Abstract

High-mobility semiconducting polymers offer the opportunity to develop flexible and large-area electronics for several applications, including wearable, portable and distributed sensors, monitoring and actuating devices. An enabler of this technology is a scalable printing process achieving uniform electrical performances over large area. As opposed to the deposition of highly crystalline films, orientational alignment of polymer chains, albeit commonly achieved by non-scalable/slow bulk alignment schemes, is a more robust approach towards large-area electronics. By combining pre-aggregating solvents for formulating the semiconductor and by adopting a room temperature wired bar-coating technique, here we demonstrate the fast deposition of submonolayers and nanostructured films of a model electron-transporting polymer. Our approach enables directional self-assembling of polymer chains exhibiting large transport anisotropy and a mobility up to 6.4 cm^2^ V^−1^ s^−1^, allowing very simple device architectures to operate at 3.3 MHz. Thus, the proposed deposition strategy is exceptionally promising for mass manufacturing of high-performance polymer circuits.

## Abstract (web summary)

Semiconducting polymers with high mobility are essential for the development of high-frequency flexible electronics, whose fabrications rely on robust printing techniques. Bucella *et al*. report the fabrication of *n*-type polymer field-effect transistors, with mobility up to 6.4 cm^2^ V^−1^s operated at 3.3 MHz, by room temperature bar-coating technique.

## Graphical abstract

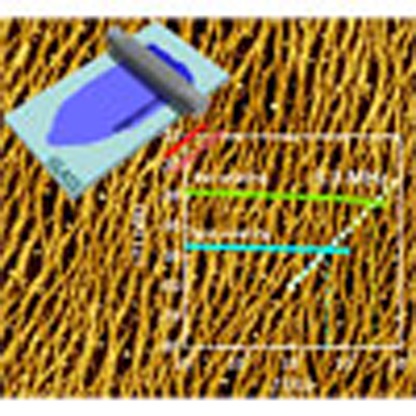

High-throughput solution-based processes for manufacturing flexible and large-area organic electronic devices^1^,^2^,^3^,^4^,^5^ are key enablers for cost-effective applications in the field of microelectronics^6^,^7^,^8^,^9^, chemical and biosensing, and in the general emerging field of wearable, portable and distributed electronics, where facile integration, mechanical flexibility and lightweight are critical advantages. Thanks to recent demonstrations of polymeric semiconductors with exceptional hole (p-type) and electron (n-type) field-effect mobilities (*μ*)^10^,^11^,^12^,^13^,^14^,^15^, now exceeding 10 and 5 cm^2^ V^−1^ s^−1^, respectively, widespread applications in such fields are possible. The most relevant figure of merit for a semiconductor, *μ*, quantifying charge transport in a field-effect transistor (FET), averages local intra- and inter-molecular transport properties typically over channels of several micrometres (usually a few tens). While it is now well established for polymeric semiconductors that an efficient short-range inter-molecular packing, favouring inter-chain electronic coupling, is necessary to achieve optimal performances^16^,^17^, directional order of the chains over large area also strongly affects charge transport performance and uniformity of the FET parameters^12^,^18^,^19^,^20^. The possibility of increasing polymer conductivity by alignment of polymer chains has been known for long time, however this has so far been achieved by means of mechanical stretching^21^ or, as in very recent reports of high-mobility semiconducting polymers, through either long post-processing techniques, such as high-temperature rubbing^22^,^23^, or through the use of confined fluid flows on pre-engraved substrates^12^. These techniques are not scalable and/or inherently slow and do not answer to the question whether a controlled and stable alignment of high-mobility polymers over large area can be achieved by means of a fast coating process, without recurring to slow post-processing steps. Therefore, it is critical to enable and control polymer film morphology and solid-state ordering over large areas, by using fast, simple and scalable techniques, compatible with high-throughput printing fabrication processes^24^.

In this work we demonstrate that the manifold molecular assembling properties of a model *n*-type polymeric semiconductor, poly{[*N*,*N*′-bis(2-octyldodecyl)-naphthalene-1,4,5,8-bis(dicarboximide)-2,6-diyl]-alt-5,5′-(2,2′-bithiophene)} (P(NDI2OD-T2)), one of the most studied *n*-type polymeric semiconductor^25^,^26^,^27^,^28^,^29^, can be finely controlled over the meso- and micro-scale for film processing at low temperatures and in ambient air using bar coating^30^. Thanks to the highly ordered elongated nanostructures, excellent electron transport properties are obtained without any pre-patterning of the substrate, neither additional, time/energy-consuming post-processing steps such as drastic thermal annealing^10^,^31^ and solvent exposure^32^. We demonstrate that bar coating enables efficient electron transport even through submonolayer polymer films (nominal thickness ∼2.2–2.4 nm), thus reducing material usage, and is a robust alternative to previous small molecule and polymeric self-assembled devices^33^,^34^. Furthermore, anisotropic transport can be achieved within the aligned microstructure of thicker (∼10 nm) bar-coated films, where the electron mobility parallel to the printing direction is >10 times that of the mobility perpendicular to the printing direction. Thus, exceptional reproducibility and data uniformity is observed for aligned films with mobilities up to 6.4 cm^2^ V^−1^ s^−1^, reaching the highest values for a polymeric *n*-type semiconductor. This result enormously facilitates the roll-to-roll compatible fabrication of printed polymer-based FETs operating in the MHz regime, mandatory for key applications such as printed radio-frequency identification tags and addressing electronics for video displays, without recurring to sophisticated processes^35^,^36^. Such level of control in the spatial alignment of nanosized supramolecular structures and in the charge transport properties over large areas, achieved with an extremely simple and high-throughput process, has strong technological relevance representing a stepping stone to the full deployment of optimal charge transport properties in mass-manufactured polymer electronics.

## Results

### Understanding the self-assembling properties of P(NDI2OD-T2)

It was recently found that in certain solvents, for example, mesitylene, P(NDI2OD-T2) strongly aggregates, and films cast from this solvent exhibit extended polymer regions, hundreds of micrometres wide, characterized by a marked molecular orientational order^37^,^38^. Thus, here we first aim at clarifying P(NDI2OD-T2) self-assembling properties and at isolating the early stages of growth of such liquid crystalline-like films, as a required rationalization step to devise an efficient printing strategy.

**Figure 1:**
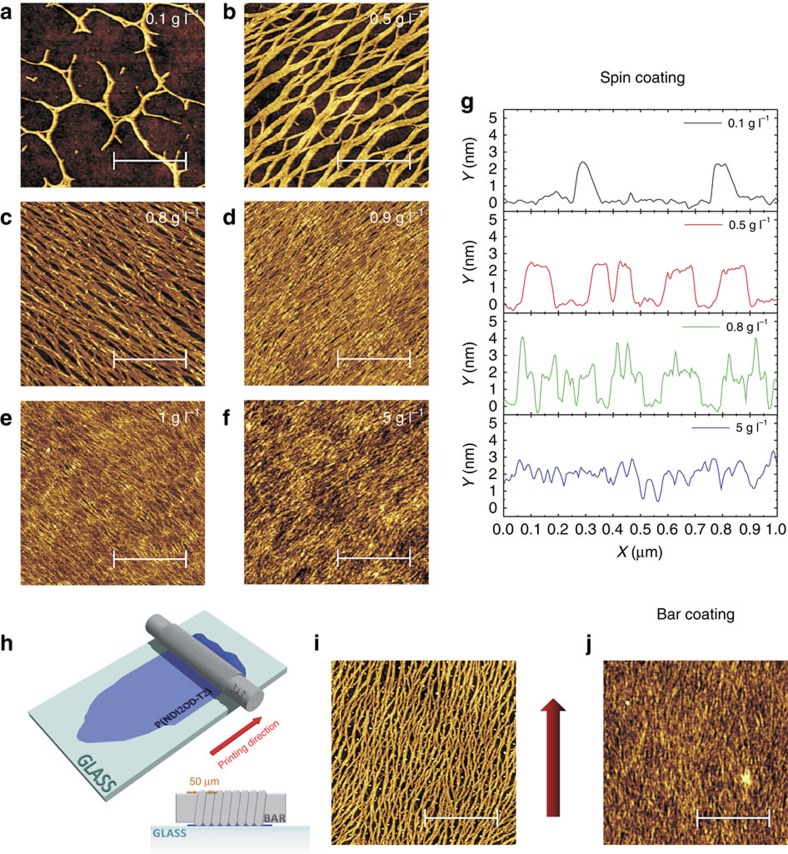
Topography of P(NDI2OD-T2) submonolayer and films deposited on glass substrates. AFM images of P(NDI2OD-T2) films spin-coated from solutions with concentration of (**a**) 0.1 g l^−1^, (**b**) 0.5 g l^−1^, (**c**) 0.8 g l^−1^, (**d**) 0.9 g l^−1^, (**e**) 1 g l^−1^ and (**f**) 5 g l^−1^. In **g** the profile analysis of **a**–**c** and **f** is reported. (**h**) Sketch of the bar-coating process. (**i**) AFM picture of a submonolayer deposited by bar-coating a 0.5 g l^−1^ solution in mesitylene. (**j**) AFM picture of a highly oriented film realized by bar-coating of a 5 g l^−1^ solution in mesitylene. Scale bars, 1 μm.

By spin-coating P(NDI2OD-T2) diluted solutions on glass, starting from a concentration of 0.1 g l^−1^ in mesitylene, elongated supramolecular structures are formed, up to 50 nm wide, with an homogenous thickness within 2.2 and 2.4 nm, corresponding to a polymer monolayer having the backbone with an edge-on orientation. Depending on the concentration and the resulting substrate coverage, the spatial arrangement of such microstructures, as observed by atomic force microscopy (AFM) images, ranges from a disordered/random pattern of branched domains (0.1 g l^−1^ in mesitylene; Fig. 1a and Supplementary Fig. 1a), to a submonolayer network. AFM pictures of submonolayers (concentration from 0.5 to 0.9 g l^−1^, Fig. 1b–d) unveil superior network connectivity along one specific direction of the substrate plane, consistently with its intrinsically anisotropic supramolecular elongated building blocks (clearly distinguishable in Supplementary Fig. 1a). Such a nicely textured molecular submonolayer denotes the selective assembling of the polymer chains during film deposition. Therefore, for these films the chains tend to assemble at the step edge of the first growing layer, exclusively contributing to its growth and limiting three-dimensional molecular growth.

By increasing the concentration above 0.8 g l^−1^, the coverage approaches full monolayer formation. It is only at this stage that additional layers start growing on top of the first layer. Contextually, a change in the self-aggregation mode occurs: the elongated nanostructures on top of the first monolayer are more clearly detached from each other, lacking an evident connectivity direction other than the main growth direction (1 and 5 g l^−1^, see Fig. 1e,f, profile analysis in Fig. 1g).

Transitions of the growth mode have been generally associated to the different surface-free energy of the hosting surfaces, since surface adhesive forces compete with the self-assembling process of the incoming molecules^39^. AFM images of the films deposited on hexamethyldisilazane-treated silicon dioxide substrates (Supplementary Fig. 1b,c) confirm that, on a lower energy surface^40^, the self-assembling of elongated and detached, quasi-1D nano-domains, prevail. Importantly, the topography of thin films (∼27 nm, Fig. 1f) deposited by spin coating from 5 g l^−1^ solutions clearly shows that the mono-directionally grown fibrillar nanostructures are sharply aligned, with optical micrograph birefringence (Supplementary Fig. 2) showing molecular alignment over a few hundreds of micrometres. A similar phenomenon can be observed by varying the film deposition methodology from spin coating to inkjet printing, as described in the supporting information (Supplementary Fig. 3).

### Large-area coating strategy

The observed film growth phenomena may enable to produce a macroscopic orientation of supramolecular fibrils, leveraging on the self-assembling properties of P(NDI2OD-T2) and on the possibility to orient polymer fibrils by controlling the dynamics of the deposition process. To achieve this goal, we have adopted a very simple wired bar-coating technique to induce a directional flow on the substrate^30^,^41^. Bar coating is a very simple, roll-to-roll compatible technique, which can be used to coat functional materials over large substrates, either rigid or flexible^42^ (for details on the technique please see Methods and Supplementary Note 1). In the reported process we adopt a bar wrapped by a wire with wire diameter of ∼50 μm (Supplementary Fig. 5) placed in physical contact with the substrate to be coated, typically an 8 × 4 cm^2^ glass slide. The deposition is performed at room temperature on a custom roll-to-roll coater by placing an excess of solution on the substrate just in front of the bar and by moving the substrate below the bar at a constant velocity of 3 m min^−1^ (Fig. 1h). In this way, only a precise amount of solution can pass through the gaps of the wire, determining the thickness of the wet film. The wet film thickness can be estimated by the geometry of the gap and, as a rule of thumb, it is 10% of the wire diameter^43^. Two different P(NDI2OD-T2) solutions having a concentration of 0.5 and 5 g l^−1^ are printed. From the 0.5 g l^−1^ solution, a submonolayer with a structure continuity along the printing direction is obtained (AFM picture in Fig. 1i and Supplementary Fig. 4). From the 5 g l^−1^ solution, ∼10 nm thick films with highly ordered quasi-monodimensional nanostructures, uniformly oriented along the printing direction, are formed (AFM picture in Fig. 1j). These results are in agreement with those observed for the spin coating and inkjet printing depositions (see Supplementary Tables 1 and 2 for details on shear rates and solutions viscosities), demonstrating that the driving force favouring alignment derives from fluid-dynamic flows occurring during the deposition, dominant over the solvent evaporation (delayed with respect to the coating and mostly developing from the border to the centre of the sample)^30^.

### Molecular and crystalline orientation of bar-coated samples

**Figure 2:**
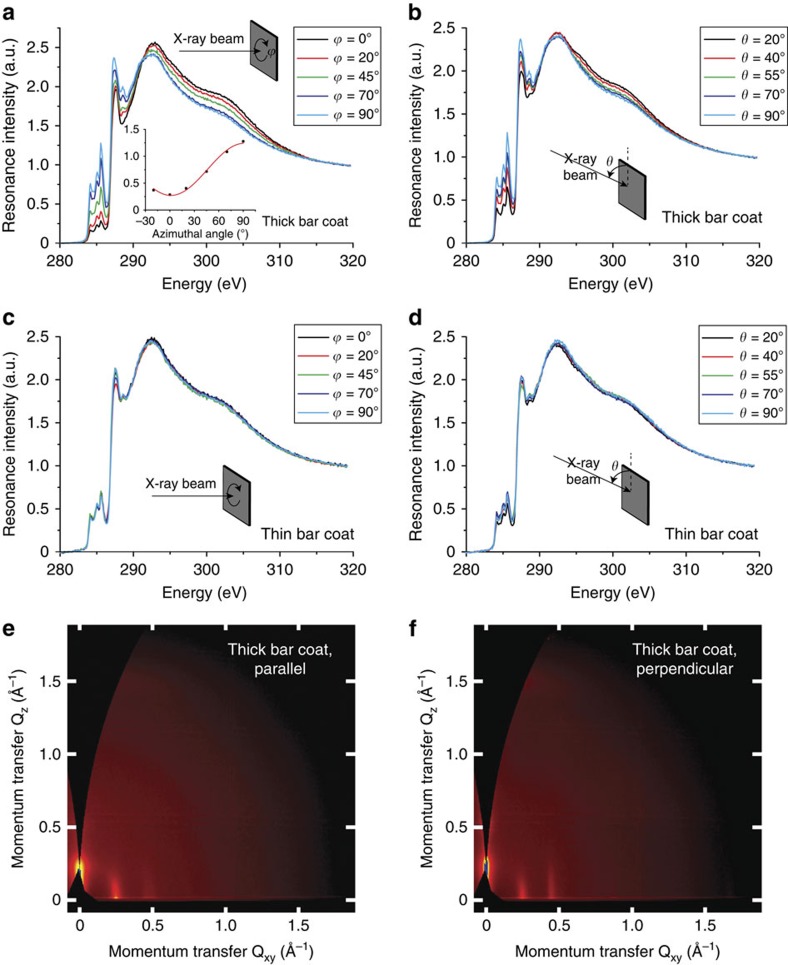
NEXAFS spectroscopy and GIWAXS patterns from bar-coated samples. (**a**,**b**) Angle-resolved NEXAFS measurements of a 10 nm (thick) bar-coated film; to probe the degree of in-plane alignment of the polymer transition dipole moments (TDMs), the NEXAFS spectra were recorded as a function of azimuthal angle, rotating the sample with respect to a normally incident X-ray beam. The TDMs of the carbon 1s → π* transitions between 284 to 286 eV are oriented perpendicular to the conjugated backbone, with the dichroism thus providing a direct measure of the degree of polymer backbone alignment at the film surface. Strong dichroism is observed for the peaks corresponding to other TDMs, for example, C 1s to C–H σ* (287.4 eV) and C 1s to C–C σ* (∼292 and 302 eV), indicating strong orientational alignment of side chains as well as the polymer backbone. (**c**,**d**) Angle-resolved NEXAFS measurements of a submonolayer (thin) bar-coated sample; (**e**,**f**) 2D GIWAXS patterns of bar-coated film (thick) recorded with the X-ray beam incident (**e**) parallel and (**f**) perpendicular to the coating direction.

Spurred by the achieved control over the polymer film topographical features, we employ synchrotron-based near-edge X-ray absorption fine-structure (NEXAFS) spectroscopy and grazing-incidence wide-angle X-ray scattering (GIWAXS) to probe the molecular and crystalline orientation of our bar-coated films (Fig. 2). NEXAFS spectroscopy probes the molecular orientation and alignment of the top few nanometres of a film, commensurate with the extent of the charge accumulation layer in top-gate FETs. Figure 2a presents the angle-resolved NEXAFS spectra (partial electron yield mode) of 10 nm thick bar-coated samples measured as a function of the azimuthal angle. Calculating a dichroic ratio as the maximum resonance intensity of the carbon 1s→π* transition divided by the minimum resonance intensity (to be consistent with the optical results below), a dichroic ratio of 4.8 is determined at the film surface. It should be noted that NEXAFS spectroscopy is equally sensitive to amorphous and crystalline polymer chains with this dichroic ratio representing an average over all chains. The tilt of the planar backbone is also characterized by performing a tilt angle scan at fixed azimuthal angle (Fig. 2b) with the observed dichroism reflecting a preferential edge-on orientation corresponding to an average tilting of the C 1s→π* transition dipole moment (TDM) of 58.7±0.5° from the surface normal.

A strong NEXAFS signal is observed also for submonolayer bar-coated samples (Fig. 2c,d), owing to the excellent surface sensitivity of NEXFAS. Very little dichroism is observed in the azimuthal scan, corresponding to a dichroic ratio of only 1.05, indicating very little, if any, long-range in-plane alignment of the polymer backbones in this case. The tilt angle scan of the submonolayer sample is consistent with a preferential edge-on orientation of the polymer backbones and consistent with the value measured for the thicker bar-coated samples. The high degree of order observed in the AFM images of the submonolayer films and the observed monolayer height are also consistent with a preferential edge-on orientation of the polymer backbone.

GIWAXS measurements of the thicker bar-coated sample (Fig. 2e,f and Supplementary Table 3) indicate a preferential face-on orientation of crystallites in the bulk, consistent with previous results where a top edge-on surface layer is observed covering predominantly a face-on oriented bulk^44^. An in-plane alkyl-stacking peak is observed at *Q*_*xy*_∼0.25 Å^−1^ with an in-plane backbone stacking peak also observed at *Q*_*xy*_∼0.45 Å^−1^, and an out-of-plane π-stacking peak at ∼1.59 Å^−1^. We use previously established methods^45^ to measure a Herman’s orientational parameter of −0.44, indicating a highly face-on oriented crystalline stacking. Comparing the alkyl-stacking scattering intensity in-plane, we find a ratio of 1.27 between parallel and perpendicular intensities, while for the backbone peak, we find a ratio of the backbone alignment peak equal to 0.13 (parallel/perpendicular). The bulk π-stacking, to which GIWAXS is sensitive, is largely oriented out of plane, so X-rays coming in both perpendicular and parallel direction scatter into the same peak, making a dichroic ratio of π-stacking from GIWAXS impossible. The higher relative intensity of the backbone peak for X-ray incidence perpendicular to the coating direction is consistent with aligned polymer backbones parallel to the coating direction and increased alkyl stacking perpendicular to it. Interestingly, this dichroic ratio of the crystalline polymer backbones determined from bulk-sensitive GIWAXS (∼7.7) is only slightly higher than the π* TDM alignment measured by surface-sensitive NEXAFS (4.8), suggesting a similar degree of backbone alignment at surface and in the bulk. However since GIWAXS only probes crystalline phases, and it is likely that amorphous domains in the bulk will not have chains well aligned, the NEXAFS dichroic ratio that averages both amorphous and crystalline chains suggest a superior degree of surface backbone alignment (consistent with observations of zone-cast PBTTT films)^46^. Comparing the bar-coated sample to a film spin-coated from mesitylene (Supplementary Fig. 13e) very similar crystallographic spacings are found indicating that films produced by both methods have the same crystal packing. The bar-coated sample however exhibits a more face-on orientation in the bulk compared to the spin-coated sample (Hermann’s parameter of −0.37) and also exhibits a slightly higher crystallinity (50% relative increase for the lamellar stacking and π-stacking directions; 120% relative increase for the backbone stacking direction).

### Electrical properties of field-effect transistors

**Figure 3:**
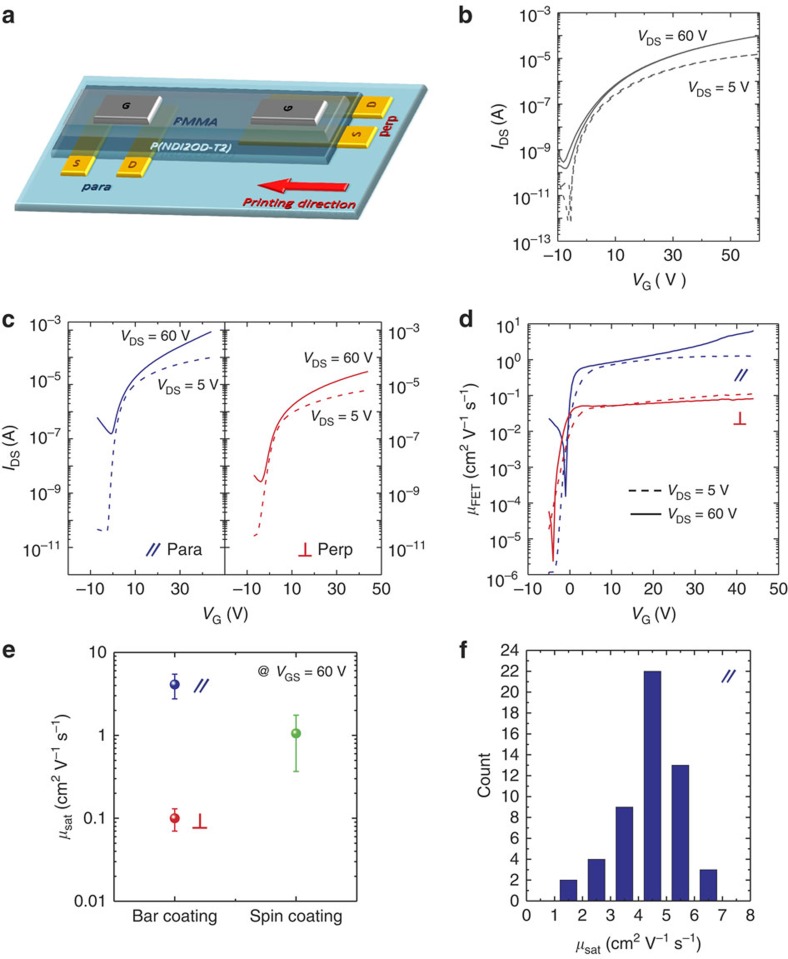
Electrical properties of FETs. (**a**) Sketch of the top gate bottom contact FETs structure employed; the probing directions are clearly represented with respect to the printing direction and indicated as para (printing parallel to the source to drain electric field) and perp (printing perpendicular to the source to drain electric field). (**b**) Typical transfer characteristic in linear (dashed) and saturation (continuous) regime of a submonolayer based FETs with *W*=2 mm and *L*=20 μm; currents ON/OFF ratio is ∼10^6^ in the linear regime. (**c**) Typical transfer characteristic plots in linear (dashed) and saturation (solid) regimes of 10 nm thick film based FETs with *W*=2 mm and *L*=20 μm with the fibrils axis oriented parallel (blue) and perpendicular (red) to the probing direction; in the linear regime, currents ON/OFF ratio is ∼10^7^ for the parallel case and ∼10^6^ for the perpendicular case. (**d**) Typical effective linear and saturation mobility as a function of the gate voltage in the perpendicular and parallel direction: the anisotropy is marked both with a high and a low lateral applied voltage. (**e**) Average saturation mobilities (circles) with their standard deviation (bars) for 56 FETs coated on an 8 × 8 cm^2^ area in both parallel and perpendicular directions, compared to a distribution obtained from 8 spin-coated FETs. (**f**) Distribution of the saturation mobility in the parallel case for 56 FETs coated on an 8 × 8 cm^2^ area.

The intriguing nanostructure of the bar-printed films lead us to study the electrical properties of both the submonolayer and the highly oriented fibrils films in top-gate bottom-contact field-effect transistors, with films printed both parallel (‘para’) and perpendicular (‘perp’) to the source to drain electric field (Fig. 3a). The transfer curve of Fig. 3b and the mobility-voltage plots of Supplementary Fig. 6 demonstrate that FETs with a submonolayer semiconductor channel exhibit ideal field-effect characteristics without significant differences along the two printing directions. This result is in agreement with AFM and NEXAFS findings of the submonolayer film structural isotropy. An average effective saturation mobility (*V*_DS_=60 V) of 0.14 cm^2^ V^−1^ s^−1^ and linear mobility (*V*_DS_=5 V) of 0.10 cm^2^ V^−1^ s^−1^ are conservatively extracted by considering the entire geometrical channel width, despite a semiconductor channel coverage of only ∼50% (Fig. 1i). These are unrivalled values for a device where the accumulated channel is confined to a single molecular strand^47^,^48^,^49^,^50^, comprising self-assembled monolayer field-effect transistors^51^,^52^,^53^, where to achieve a mobility of 0.08 cm^2^ V^−1^ s^−1^ the use of specific anchoring groups is required.

FETs fabricated with the thicker printed films show instead a marked charge transport anisotropy both in saturation and in linear regimes (Fig. 3c,d and Supplementary Fig. 12). Devices measured in saturation (*V*_DS_=60 V) exhibit an *I*_DS_ difference of 35 × whereas 15 × is the difference in the linear currents (*V*_DS_=5 V). This result clearly demonstrates a more favourable electronic transport along the fibrils axis, parallel to the molecular backbone. Note that FETs fabricated by bar-coating a polymer solutions in ortho-dichlorobenzene (oDCB), a solvent with a poorer pre-aggregating effect, do not display transport anisotropy and show in both parallel and perpendicular directions currents similar to the ones obtained for mesitylene in the perpendicular case (Supplementary Fig. 7).

**Figure 4:**
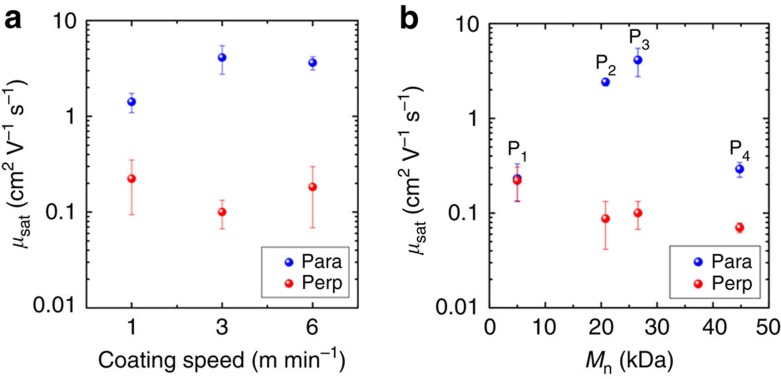
Effect of coating speed and of molecular weight. (**a**) Saturation mobility (*V*_GS_=*V*_DS_=60 V) for FETs fabricated by bar-coating at different velocity (1, 3 and 6 m min^−1^) a solution of P(NDI2OD-T2) with *M*_n_ of 26.6 KDa and PDI of 3.2, probing the transport parallel (black dots) and perpendicularly (red dots) to the coating direction. The solution concentration is 5 g l^−1^ in mesitylene. (**b**) Saturation mobility (*V*_GS_=*V*_DS_=60 V) for FETs fabricated by bar-coating solutions of P(NDI2OD-T2) with different *M*_n_ and PDI, probing the transport parallel (black dots) and perpendicularly (red dots) to the coating direction. Solution concentration (5 g l^−1^ in mesitylene) and coating speed (3 m min^−1^) were kept constant.

To test the effect of the coating speed on the electrical properties of bar-coated thin films, we perform coating at different speeds, including 1, 3 and 6 m min^−1^. The corresponding FETs data are reported in Fig. 4a. In all cases it is possible to achieve alignment and to observe transport anisotropy. In the case of the slower speed of 1 m min^−1^, the anisotropy is the weakest (*μ*_sat-para_/*μ*_sat-perp_=6.4) and the average saturation mobility in the parallel case is only *μ*_sat-para_=1.41 cm^2^ V^−1^ s^−1^, indicating that the alignment is not as efficient as at 3 m min^−1^, likely owing to a reduced shear. A faster coating speed of 6 m min^−1^ produces an anisotropy which is only slightly inferior to the 3 m min^−1^ case (*μ*_sat-para_/*μ*_sat-perp_=20.1), and where the average saturation mobility in the parallel case reaches *μ*_sat-para_=3.62 cm^2^ V^−1^ s^−1^. To test also the effect of molecular weight, we perform coating tests with a series of batches with the following average molecular weight, *M*_n_ (and polidispersity, PDI): 5 kDa (1.8), 20.8 kDa (2.4), 26.6 (3.2), 44.8 kDa (2.6) (in Fig. 4 referred as P_1_, P_2_, P_3_ and P_4_, respectively), while keeping constant the solution concentration (5 mg ml^−1^ in mesitylene) and the coating speed (3 m min^−1^). The results, in terms of saturation mobility for devices probing the transport parallel and perpendicularly to the coating direction, are reported in Fig. 4b. These results show that a very low *M*_n_ of 5 kDa does not allow observing any anisotropy, as an effect of the impossibility to align the polymer backbones. This is likely due to an ineffective aggregation in mesitylene solution, as evidenced by ultraviolet–vis spectra (Supplementary Fig. 8). This results also in a poor average mobility of 0.23 cm^2^ V^−1^ s^−1^. The alignment, and consequent anisotropy, is very effective with *M*_n_=20.8 kDa (PDI=2.4) and *M*_n_=26.6 kDa (PDI=3.2). Between the two, the 26.6 kDa batch shows the best results. However, since the *M*_n_ are very close, we cannot exclude an effect of the different PDI of the two batches. When we adopt consistently higher *M*_n_, as in the case of the 44.8 kDa batch, we enter again a regime where alignment is problematic, likely owing to a too strong entanglement of the chains. In this case we do observe a small anisotropy, but the average mobility in the parallel case is limited to 0.29 cm^2^ V^−1^ s^−1^.

To prove the suitability of the deposition method for large area, we process the semiconductor over an area of 8 × 8 cm^2^ comprising 56 devices for both coating directions. For the parallel case we obtain an average saturation mobility 
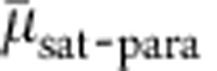
 of 4.1±1.4 cm^2^ V^−1^ s^−1^, with a maximum *μ*_sat-para_=6.4 cm^2^ V^−1^ s^−1^, reaching the highest values reported so far for an n-type polymer, and an exceptional average linear mobility 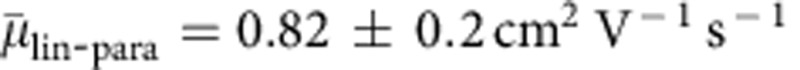, with a maximum *μ*_lin-para_=1.2 cm^2^ V^−1^ s^−1^ (intrinsic linear mobility values, i.e. mobility values extracted without the effect of Contact Resistance are reported in Supplementary Fig. 12). Such maximum linear mobility, achieved at far lower lateral fields (∼2.5 kV cm^−1^) than those in saturation and in a regime with a very weak gate voltage dependence (Fig. 3d), is remarkable and represents a record value for a solution-processed organic n-type semiconductor, even considering single crystal transistors^54^,^55^,^56^,^57^,^58^. On the same 8 × 8 cm^2^ area, for the perpendicular case we recorded an average saturation mobility 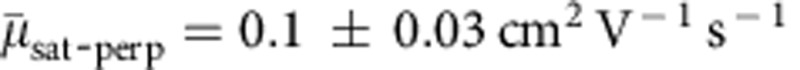, and an average linear mobility 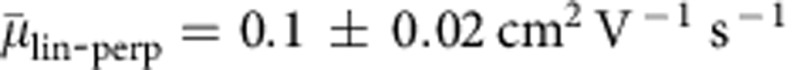 (the full statistics for both distributions are reported in Supplementary Figs 9 and 10).

It is also noteworthy that spin-coated films deposited from mesitylene, a medium in which solvent-induced fibrillar macro-domains are still present but randomly oriented along the whole surface, display an average mobility 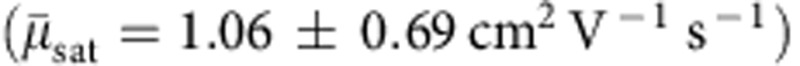, which is in between those measured for the bar-coated films printed in the parallel and perpendicular directions with a clearly broader distribution of mobility values (Fig. 3e,f and Supplementary Fig. 11). Much lower relative standard deviations of 30 and 33% are calculated in the parallel and perpendicular directions, respectively, with respect to the 63% for the spin-coated semiconductor-based devices. Therefore we can state that an effective, yet simple, control of the morphology realized here, not only achieves the highest mobility range, but also enables a stronger reproducibility and uniformity of the transport properties, two aspects of the utmost importance for large-area manufacturing of devices.

### Unveiling the functional microstructure of bar-coated films

**Figure 5:**
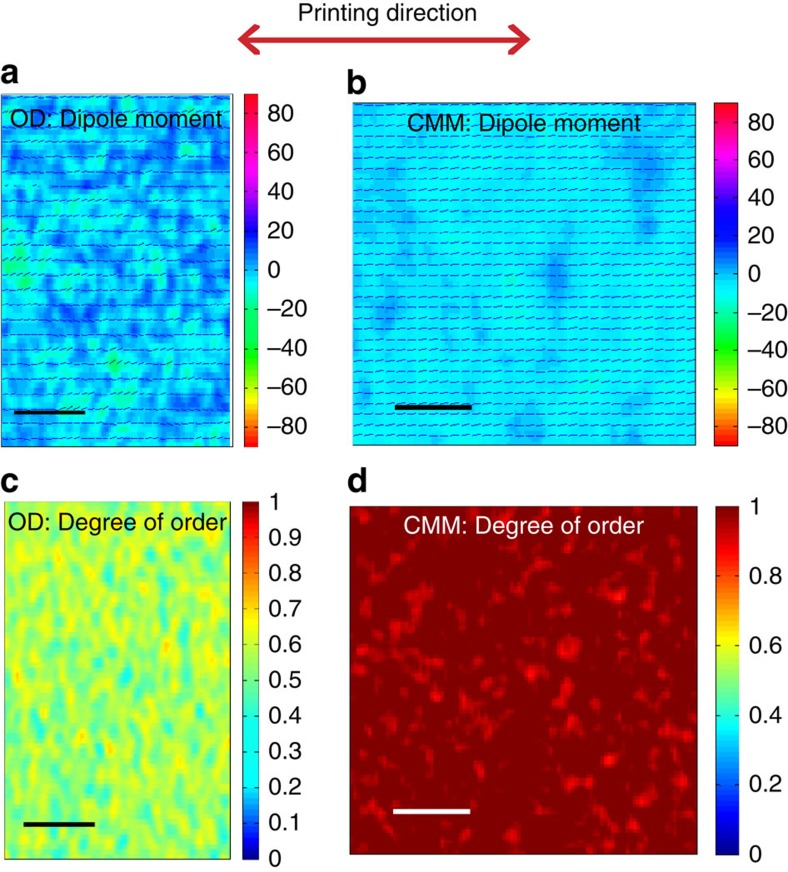
Polarized optical density and charge modulation microscopy maps. 13 × 24 μm^2^ polarized optical density (OD) map (**a**) and 18 × 18 μm^2^ CMM map (**b**) with the indication of the polymers backbone orientation (black dashed lines) and the relative degree of orientational order maps (**c**,**d**) of a bar-coated film. On the top of the picture the direction of printing with respect to the maps is reported. Scale bars, 4 μm. The mean DR values are calculated relative to these scanned areas.

Interestingly, the large observed anisotropy for the bar-coated films is not accompanied by a significant optical dichroic ratio (DR), as measured by polarized ultraviolet–vis absorption spectroscopy (never superior to 1.8, Supplementary Fig. 14). This is in contrast to general thinking and it is far less intuitive to rationalize than in more highly optically aligned samples^22^. A possible way to solve this apparent discrepancy is to hypothesize a superior chain alignment of the film polymer surface with respect to the bulk, which would be consistent with the structural data and with a previous study confirming a different surface structure in P(NDI2OD-T2) films^44^. Thus, we utilized polarized charge modulation microscopy (p-CMM)^59^,^60^, which maps the TDM of specific charge-induced transitions, and relates it to the preferential in-plane alignment of the polymer backbone. Being selective to charge-induced features, p-CMM is sensitive only to the conjugated segments that are involved in the charge transport. p-CMM maps can further be compared with polarized confocal microscopy (pCM) maps, providing instead information of the average alignment of all conjugated segments of the film. By adopting a polarized beam at 690 nm, close to the main optical absorption peak of P(NDI2OD-T2) in the visible (Supplementary Fig. 14), maps of the orientation (Fig. 5a,b) and of the Degree of orientational Order (DO; Fig. 5c,d) of the TDM from pCM (Fig. 5a,c) and p-CMM (Fig. 5b,d) are measured in the same channel area of a working FET. In P(NDI2OD-T2) the dipole moment of the electronic transition probed at 690 nm oscillates parallel to the polymer backbone^60^, therefore maps in Fig. 5a,b are directly informative of the polymer backbone orientation within the film. Figure 5a confirms that a clear preferential alignment of the polymer backbones along the printing direction occurs, with a mean DO 
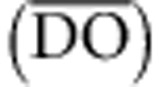
 of 56%. From the 
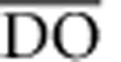
 value, the mean optical DR 
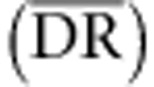
 can be extracted using the formula 
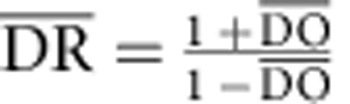
, resulting in a value of 3.5 for pCM. Already by comparing the maps in Fig. 5a,b it can be observed that the conjugated segments probed with p-CMM appear to be more finely aligned along the coating direction. Quite remarkably, for p-CMM a 
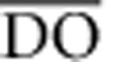
 value of 98% can be extracted, corresponding to a very high 
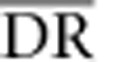
 of 99, univocally rationalizing the observed transport anisotropy. Such a high DR is only in part ascribable to a superior molecular alignment at the top semiconductor surface, since it is much larger than the value of 4.8 obtained by surface-sensitive NEXAFS. Therefore we can conclude that the high level of structural alignment is a prerogative of the P(NDI2OD-T2) chain fraction solely probed by charge accumulation, which effectively selects energetic sites within the most orientationally ordered phase of the channel. Thanks to p-CMM we have therefore unveiled the functional microstructure of bar-coated films, a very limited portion of conjugated segments at the interface with the dielectric which is responsible for the observed charge transport properties and tend to elude common structural investigations which are not selective to charged molecules.

### Maximum operational frequency of bar-coated FETs

**Figure 6:**
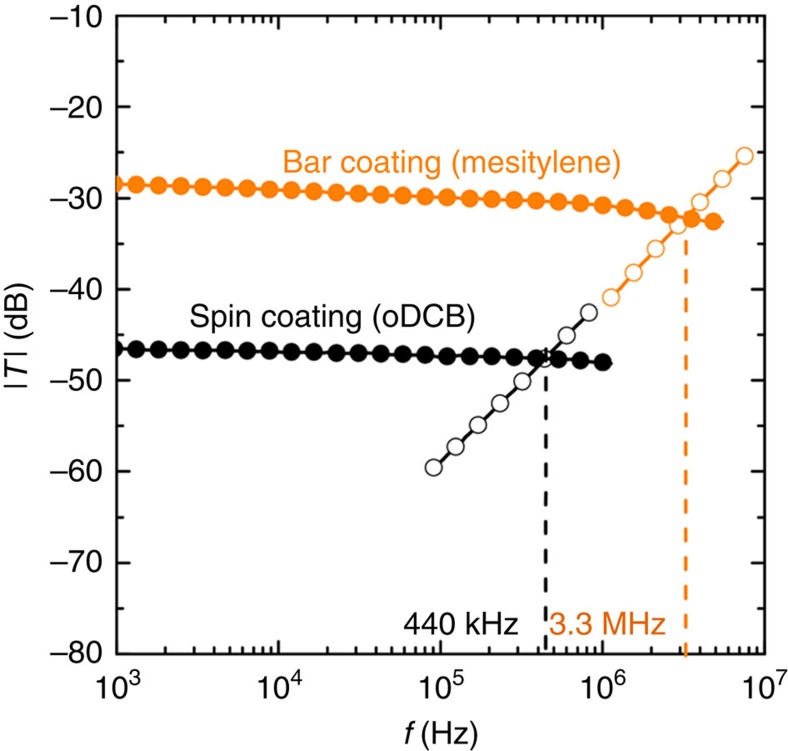
Frequency of transition of FETs. Frequency characterization of devices made by a standard spin-coating of a 5 g l^−1^ solution of P(NDI2OD-T2) in oDCB (black symbols) and a bar-coating of a 5 g l^−1^ solution of the same polymer in mesitylene (orange symbols). The input/output ac voltage transfer function has two contributions: the channel signal (filled symbols), which depends on the transconductance of the FET and the gate capacitance signal (open symbols), owing to the capacitive current that flows through the gate. The intersection between the two contributions results in the transition frequency *f*_t_, above which the capacitive coupling dominates over the channel contribution. The devices have *W*=200 μm and *L*=5 μm.

The technological relevance of these results can be assessed by evaluating the transition frequency *f*_t_ (refs 61, 62, 63), that is, the maximum operational frequency, of printed polymer FETs (see Methods, Supplementary Method 1 and Supplementary Figs 16–19 for details). These FETs are fabricated by bar-coating P(NDI2OD-T2) from mesitylene perpendicularly to the electrodes defining a channel length (*L*) of 5 μm and biased in the saturation regime and compared with standard devices, made by spin coating the same semiconductor from oDCB within an identical device architecture (Supplementary Fig. 15). The device realized by spin-coating achieves *f*_t_=440 kHz, while an impressive *f*_t_=3.3 MHz is measured for the bar-coated one, a remarkable value for devices which are not optimized at all for high-frequency operation (Fig. 6). An *f*_t_ >3 MHz is the highest reported to date for FETs with a printed organic semiconductor^35^,^64^,^65^,^66^, and it has been obtained without recurring to drastic device downscaling, a channel length of 5 μm being achievable with printing techniques such as reverse off-set^67^ and femtolitres gravure printing^66^, nor to any self-aligned architecture.

## Discussion

By understanding and exploiting the one-dimensional self-assembly of P(NDI2OD-T2), highly controlled printed submonolayers and anisotropic thin films with excellent transport properties are demonstrated. In particular, bar-coated submonolayer FETs with homogenous semiconductor thickness exhibit the highest performance so far achieved for such molecular thick, solution-processed structures (*μ*_sat_≈0.14 cm^2^ V^−1^ s^−1^). Moreover, by controlling the ink flow directionality with a bar-coating deposition technique, shear-aligned thin films with a highly oriented functional surface are realized, without the need for additional post-processing steps. This approach produces a marked FET mobility anisotropy and greater performance uniformity with respect to the spin-coating deposition, and excellent electron mobility along the printing direction, with a remarkable maximum value of 6.4 cm^2^ V^−1^ s^−1^ and an average value of 4.1±1.4 cm^2^ V^−1^ s^−1^. The latter value represents the highest electron mobility so far reported for P(NDI2OD-T2) and among the highest for n-type organic semiconductors in general. Critical to the achievement of such result is the deviation from common bulk alignment processes, but the use of a very simple, low-cost and roll-to-roll compatible printing technique in combination with suitably aggregating solvents. Thus, our approach enables to control the functional morphology within a few molecular layers from the dielectric surface in a FET, as evidenced by charge modulation microscopy investigations, and revealing an exceptionally high dichroic ratio of 99 for charge-induced features in the fraction of conjugated segments contributing to charge transport. The mere adoption of this fast coating approach allows to strongly enhance the highest operational frequency of FET devices, achieving a transition frequency of 3.3 MHz, the highest so far reported for a printed polymer, with a very basic device architecture characterized by channel length achievable through printing and unoptimized for high-frequency operation. Such operation frequency therefore holds great promises for the adoption of the proposed semiconductor deposition strategy for the development of large-area fast-printed polymer circuits with improved computation capabilities and fast driving properties and it paves the way for their profitable use in radio-frequency identifications tags, in row and column drivers for fast detector arrays and video displays. At the same time it expands the maximum bandwidth achievable for printed polymer front-end analogue circuits, for example, amplifiers integrated in printed sensors, and mixed-signal circuits, such as analogue-to-digital converters, essential building blocks for signal conditioning in wearable and distributed sensors.

## Methods

### Samples and devices fabrication

Low alkali 1737F Corning glasses or SiO_2_ were used as substrates for films and devices realized in this work. The substrates were standard cleaned in ultrasonic bath of Milli-Q water, acetone and isopropyl alcohol respectively and exposed to O_2_-plasma at 100 W. The hydrophobic treatment of the SiO_2_ was performed by exposing the surface to vapours of hexamethyldisilazane for a night. P(NDI2OD-T2) was purchased from Polyera Corporation (Activink N2200). The data here reported refer to a batch with an average molecular weight *M*_n_ of 26.6 kDa and a PDI of 3.2. Solutions of 0.01, 0.05, 0.1, 0.5, 1 and 5 g l^−1^ in mesitylene were prepared and filtered by 0.2 μm PTFE filters. The samples for AFM characterization and X-ray analysis were prepared by three different deposition methods: by spin-coating all the solutions at 1,000 r.p.m. for 30 s in nitrogen atmosphere, by inkjet printing the 5 g l^−1^ solution with a Fujifilm Dimatix Materials Printer DMP-2831 (1 pl drops volume cartridge) in air condition and by bar-coating the 0.5 and 5 g l^−1^ solutions in air condition. We adopted a stainless steel TQC spiral bar applicator with an application length of 320 mm wrapped with a stainless steel wire of 50 μm of diameter. We suspended the target substrate on a plastic web of a custom roll-to-roll coater and we deposited 100 μl of solution on the substrate in front of the bar. A wet film was formed at room temperature by moving the substrate below the bar at different coating speeds of 1, 3 and 6 m min^−1^. The evaporation of the solvent occurs on a time scale of 30 s. FETs with top-gate, bottom staggered contacts were fabricated. Bottom electrodes were patterned by a lift-off photolithographic process and deposited by evaporation of a 1.5 nm thick Cr adhesion layer and 15 nm thick Au film. Patterned substrates were cleaned by ultrasonic bath in isopropyl alcohol for 2–3 min and exposed to O_2_-plasma at 100 W for 10 min before the deposition of the semiconductor and the dielectric layers. After the deposition of the semiconductor, the devices were annealed on a hot plate for 14 h at 120 °C in nitrogen atmosphere. Poly(methyl methacrylate) (PMMA) (Sigma-Aldrich) with *M*_w_=120 kg mol^−1^ was spun from *n*-butyl acetate (with a concentration of 80 g l^−1^, for devices with *L*=20 μm, and 60 g l^−1^, for the *L*=5 μm devices). Dielectric layers with thickness of 600 and 300 nm were obtained. After the dielectric deposition, the devices were annealed under nitrogen, on a hot plate, at 80 °C for 2 h. Thick Al (50 nm) was thermally evaporated through a shadow mask to define gate contacts. For p-CMM measurements, the perfluorinated polymer CYTOP CTL-809M dielectric (Asahi Glass) was spun as received at 4,000 r.p.m. for 90 s (film thickness, 550 nm) as the dielectric layer, and thermally evaporated 4.5 nm thick Au semi-transparent gate electrodes were employed. For the frequency characterization of the devices, poly(3,4-ethylenedioxythiophene) polystyrene sulfonate (PEDOT:PSS) gate electrodes were patterned with inkjet printing to overlap only the portion of electrodes that delimited the channel.

### Film morphology characterization

The surface topography of the films was measured with an Agilent 5500 Atomic Force Microscope operated in the acoustic mode. GIWAXS patterns were collected at the SAXS/WAXS beamline at the Australian Synchrotron^68^ using a Pilatus 1 M photon detector. Photons (9 keV) were aligned parallel to the surface of the samples using a crystal analyser. Scattering patterns were collected at incident angles ranging from 0.05 to 0.25°, with the images reported taken at the critical angle identified by the angle with the highest scattering intensity. NEXAFS spectra were collected at the SXR beamline of the Australian Synchrotron^69^ and analysed using previously used protocols^44^.

### Electrical and CMM characterization

The electrical characteristics of transistors were measured in a nitrogen glovebox on a Wentworth Laboratories probe station with a semiconductor device analyser (Agilent B1500A). The linear and saturation mobility values were calculated using the gradual-channel approximation. The mobility data reported do not take into account contact effects, that is, the data correspond to an effective mobility. The contact resistance, extracted from a careful study for devices with a channel length of 5, 10 and 20 μm (reported in detail in the Supplementary Note 2) is limited, ∼1.8 kΩ cm and ∼12 kΩ cm in the parallel and perpendicular case, respectively. Therefore the effective mobility is very close to the intrinsic mobility for all characterized devices. The frequency response was investigated by a network analyser (Agilent ENA series) with a signal modulate in the range 5–3 × 10^8^ Hz and with a pick-to-pick amplitude of 720 mV, forcing the dc bias with a source metre (Agilent B2912A precision source/measure unit). Further details are reported in Supplementary Method 1. The CMM data were collected with a homemade confocal microscope in transmission configuration using a light source consisting in a supercontinuum laser (NKT Photonics, SuperK Extreme) monochromated in the 500–1,000 nm region with linewidths between 2 and 5 nm. The sample was kept in a nitrogen chamber. The gate voltage of the transistor was sinusoidally modulated at 989 Hz between 20 and 60 V, with a waveform generator (3390, Keithley) amplified by a high-voltage amplifier (WMA- 300, Falco Systems), while the source and drain contacts were kept at short-circuit.

## Additional information

**How to cite this article:** Bucella, S. G. *et al*. Macroscopic and high-throughput printing of aligned nanostructured polymer semiconductors for MHz large-area electronics. *Nat. Commun.* 6:8394 doi: 10.1038/ncomms9394 (2015).

## Supplementary Material

Supplementary Information Supplementary Figures 1-20, Supplementary Tables 1-3, Supplementary Notes 1-2, Supplementary Methods and Supplementary References
