# Supplementary material for: Macroscopic and high-throughput printing of aligned nanostructured polymer semiconductors for MHz large-area electronics
Source: Nat Commun. 2015 Sep 25;6:8394. doi: 10.1038/ncomms9394 (PMC4596007; doi:10.1038/ncomms9394)
Supplement: Supplementary Information — Supplementary Figures 1-20, Supplementary Tables 1-3, Supplementary Notes 1-2, Supplementary Methods and Supplementary References [file ncomms9394-s1.pdf]

Supplementary Figures

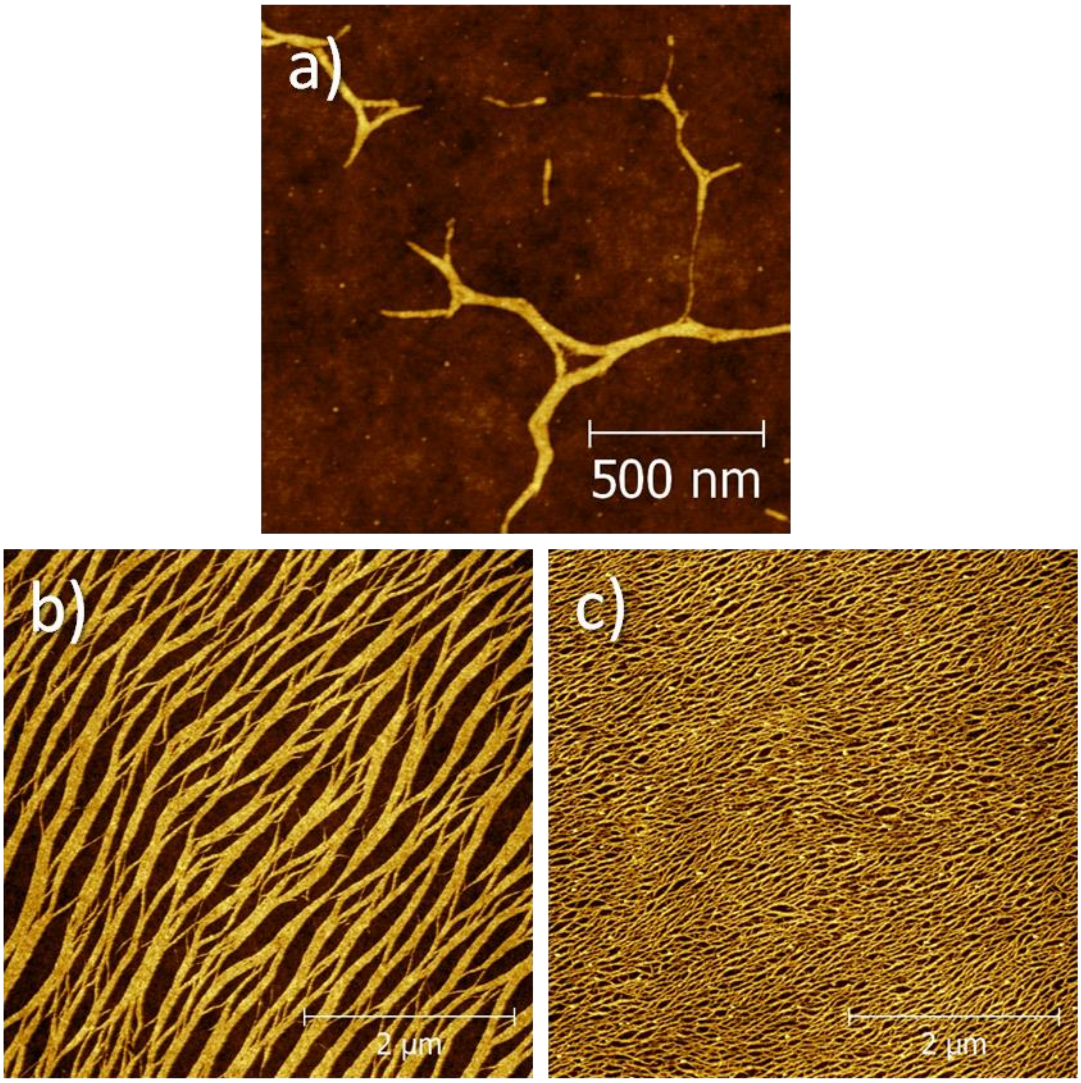

Supplementary Figure 1|AFM images of P(NDI2OD-T2) submonolayers and thin films. a) AFM picture of a branched aggregate of P(NDI2OD-T2) deposited by spin-coating from a 0.05 g l^{-1} solution in mesitylene. b,c) AFM pictures of spun P(NDI2OD-T2) films, from solution with concentration of 0.5 g l^{-1} in mesitylene, on b) silicon oxide after an oxygen plasma treatment and c) after Hexamethyldisilazane (HMDS) treatment.

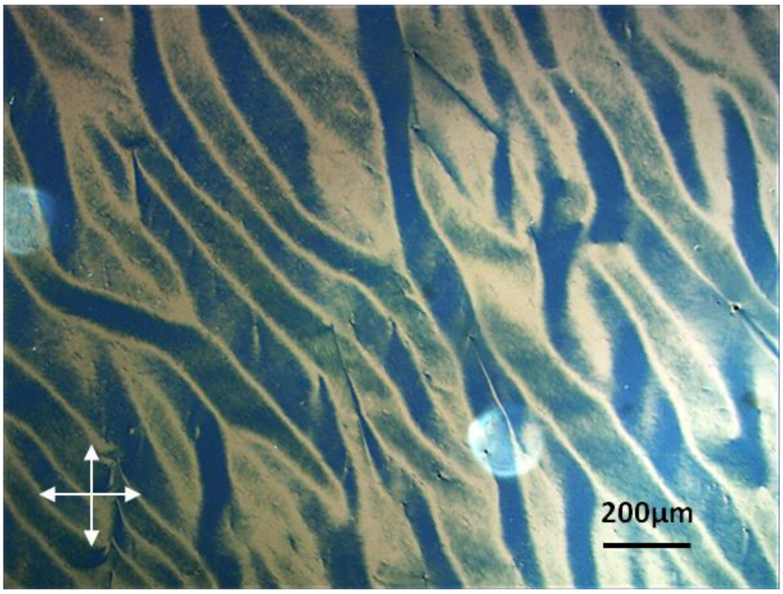

Supplementary Figure 2|Birefringence in P(NDI2OD-T2) films. Optical microscope image taken through two crossed-polarizers to highlight birefringence in a P(NDI2OD-T2) film deposited by spin-coating from 5 g l^{-1} solution in mesitylene. The dark areas represent the polymer backbone alignment in one of the two light polarizing directions. The polarized optical microscope image was measured in transmission mode with a Zeiss Axio Scope A1 equipped with two crossed polarizers.

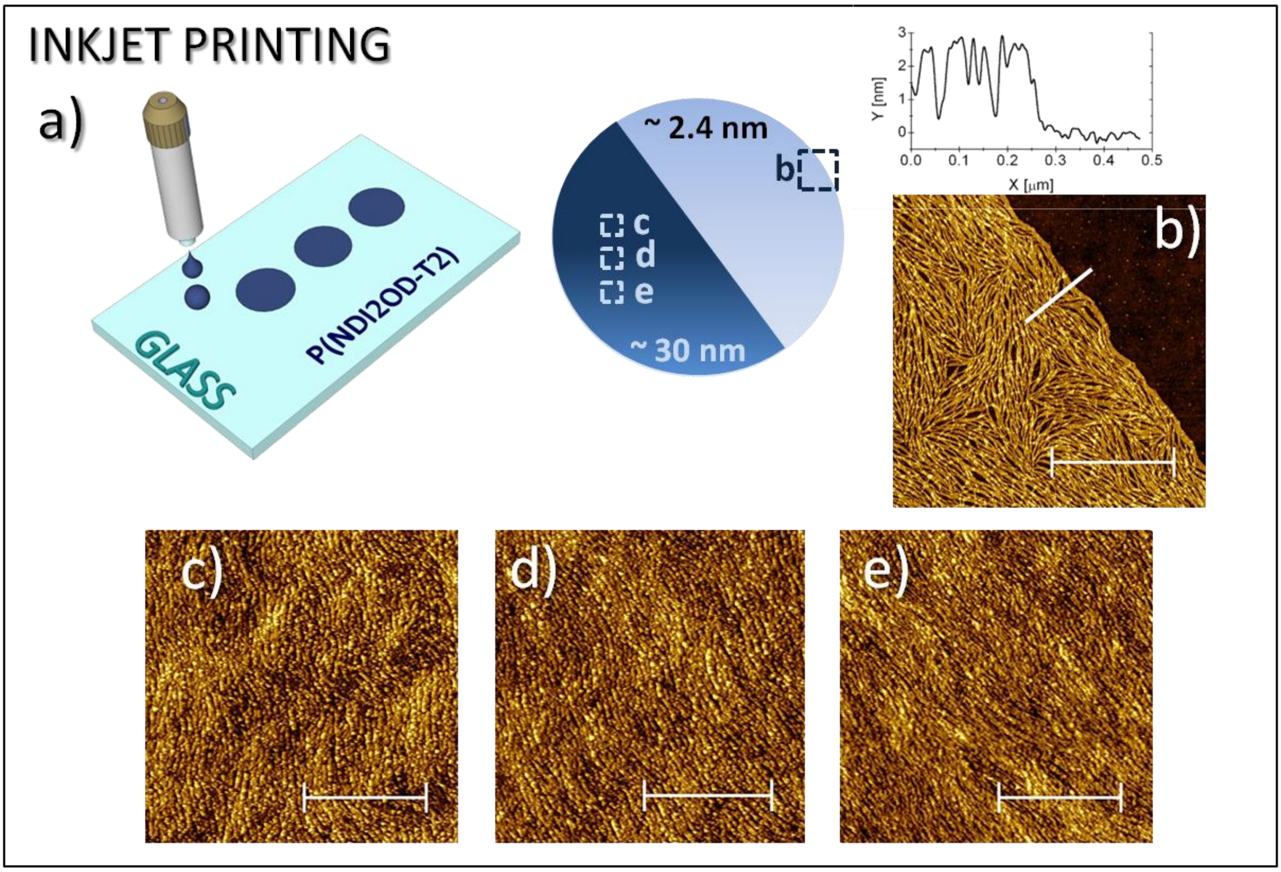

Supplementary Figure 3|Inkjet printed submonolayers and films. a) Sketch of the inkjet-printing process for the deposition of disk-shape films. b) A ~30 μm disk-shaped ~2.4 nm thick submonolayer is formed when inkjet printing a single 1 pl droplet, 5 g l^{-1} in mesitylene (see the profile); only at the disk edge fibrils do align along the contact line, owing to a pinning induced confinement. In contrast, in the inner part of the disk no preferential alignment is observed, consistently with surface adhesion forces dominating the layer deposition, rather than solvent evaporation. From c) to e) AFM pictures taken from three different positions of a ~30 nm thick disk-shape film obtained by multiple jetting in the same position; similarly to spin coating results, thicker inkjet printed films result in a fibrillar topography and in a long-range alignment, in this case forming concentric rings. During the film formation a clear drop de-pinning occurs when the first layer(s) is(are) formed, and the contact-line freely flows toward the center during the drying. The ring-like alignment of supramolecular units is most likely connected to the circular shape of the drop edge along with the contact-line dynamics during the evaporation.

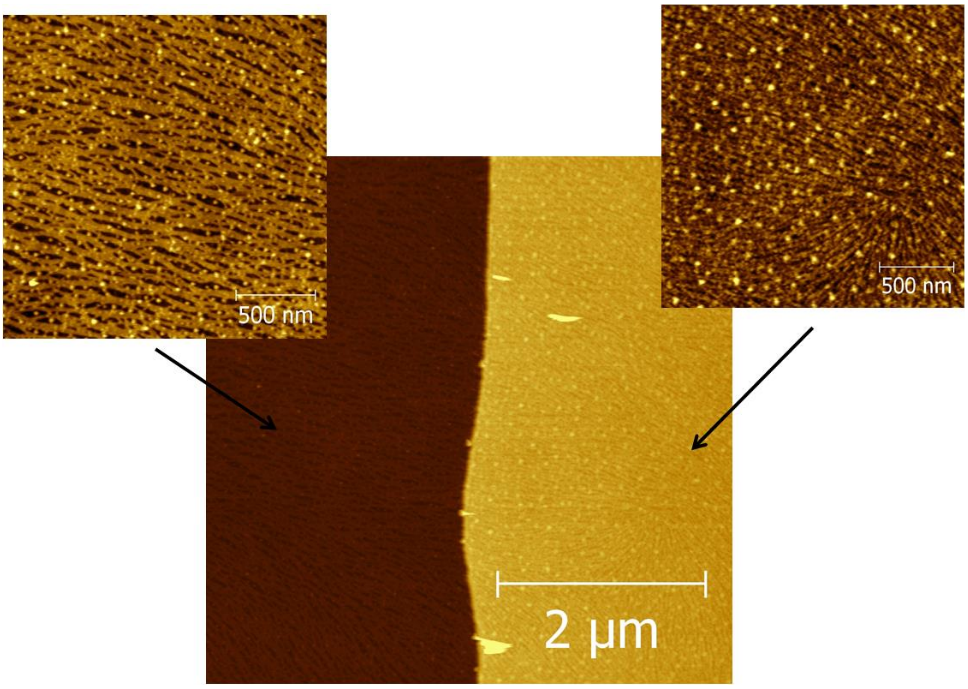

Supplementary Figure 4|Bar-coated submonolayers. Bar-coated submonolayer on a plasma treated glass substrate (on the left) with gold patterned electrodes (on the right).

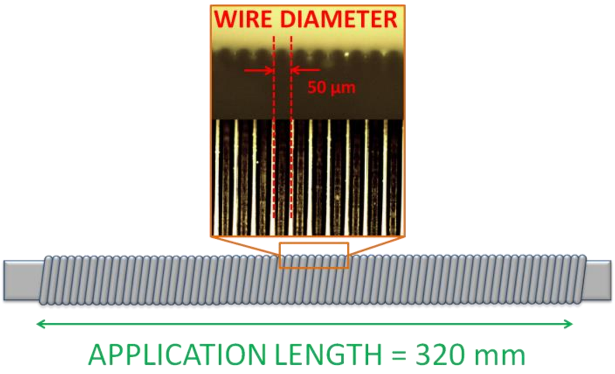

Supplementary Figure 5| Sketch and optical microscopy picture of the adopted bar.

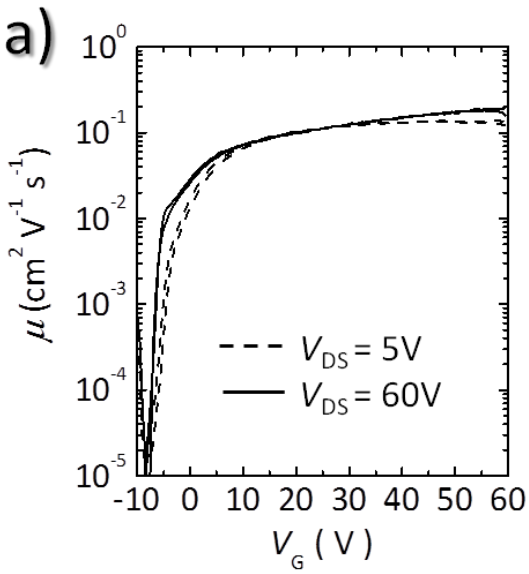

Supplementary Figure 6|Isotropic transport properties in submonolayer based FETs. a) Effective linear and saturation mobility as a function of the gate voltage for a submonolayer based device. b) Average saturation mobility and standard deviation calculated from 8 devices for both coating directions.

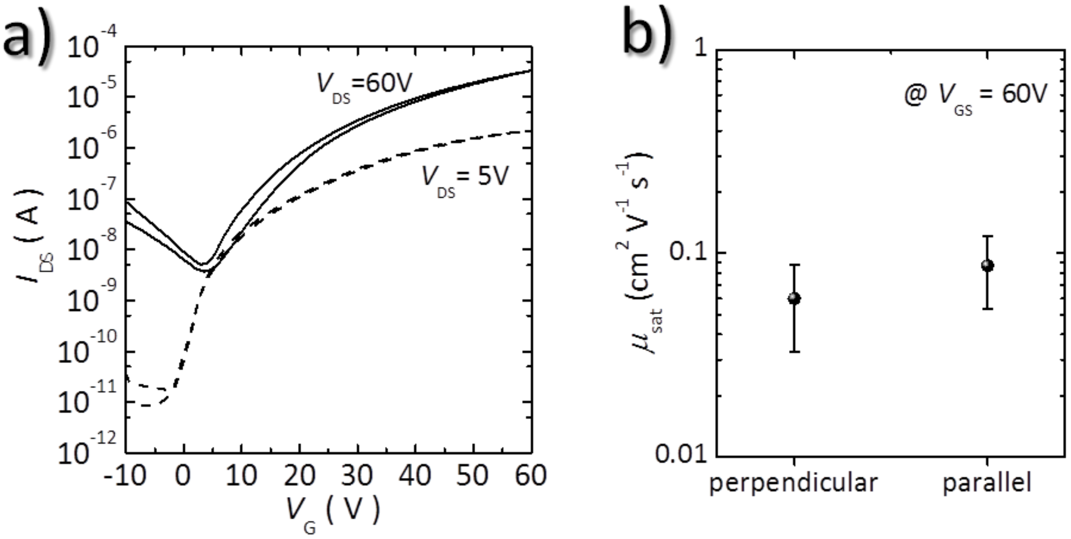

Supplementary Figure 7|Electrical properties of oDCB bar-coated FETs. To stress on the role of the pre-aggregating solvent in the fibrillar ordering, FETs with a ~30 nm semiconductor thick film bar-coated from a solution in ortho-dichlorobenzene (oDCB), a solvent with a poorer pre-aggregating effect, were also fabricated as reference samples. a) Transfer characteristics in linear

and saturation regime measured on an FET made by bar-coating a solution of P(NDI2OD-T2) of 5 gl^{-1} in oDCB. b) Average saturation mobility and standard deviation calculated for 8 devices for both coating directions. Such films show a scant anisotropy of the transport, with mobility values of 0.06 ± 0.03 $cm^2 V^{-1} s^{-1}$ in the perpendicular and 0.09 ± 0.03 $cm^2 V^{-1} s^{-1}$ in the parallel direction, consistently with a much weaker alignment of the 1D nanostructures on a large area.

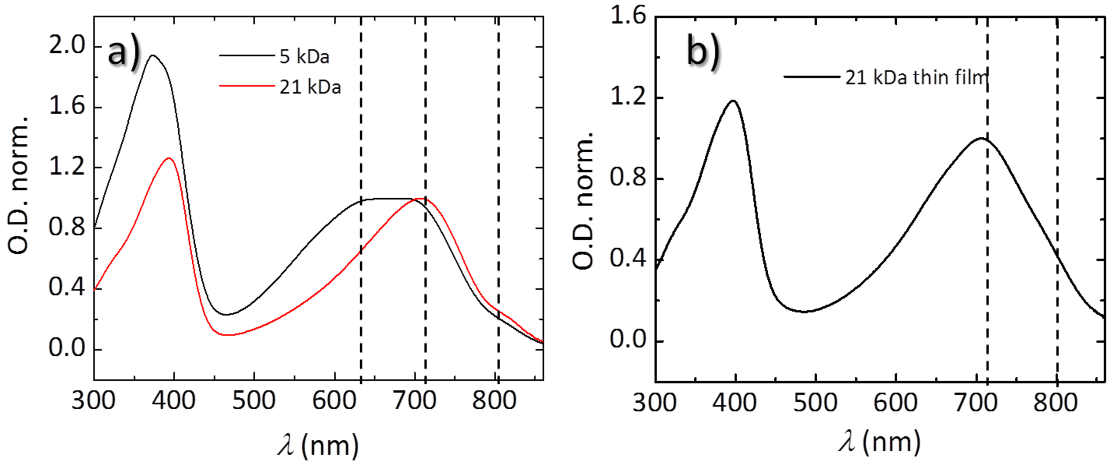

Supplementary Figure 8 | Optical absorbance of P(NDI2OD-T2) solutions and films. a) UV-vis spectroscopy of P(NDI2OD-T2) as a function of M_n in mesytilene. Both polymers show two bands attributed to the $\pi - \pi$ * transition, the high energy band (≈ 350 – 450 nm) is assigned to excited states more localized on the thiophene units whereas the low energy broad band (≈ 550 – 850 nm) is assigned to excited states with a prevalent HOMO–LUMO contribution and attributed to charge transfer (CT) states. The main transition at ≈ 700 nm and the shoulder at ≈ 800 nm have been recently assigned to an aggregate effect, while the contribution of the band at ≈ 600 nm is associated to not aggregated molecules. The polymers with M_n = 21 kDa displays a neat prevalence of aggregates absorption and in total agreement with literature.[1,2] Differently, in the spectra of polymers with M_n = 5 kDa, a clear contribution of not-aggregated polymers is still visible, also reflected by the higher relative intensity of the high energy bands.[3] b) UV-vis spectrum of P(NDI2OD-T2) film from 21 kDa polymer in mesitylene solution. Spectral features are identical to those observed in solution.

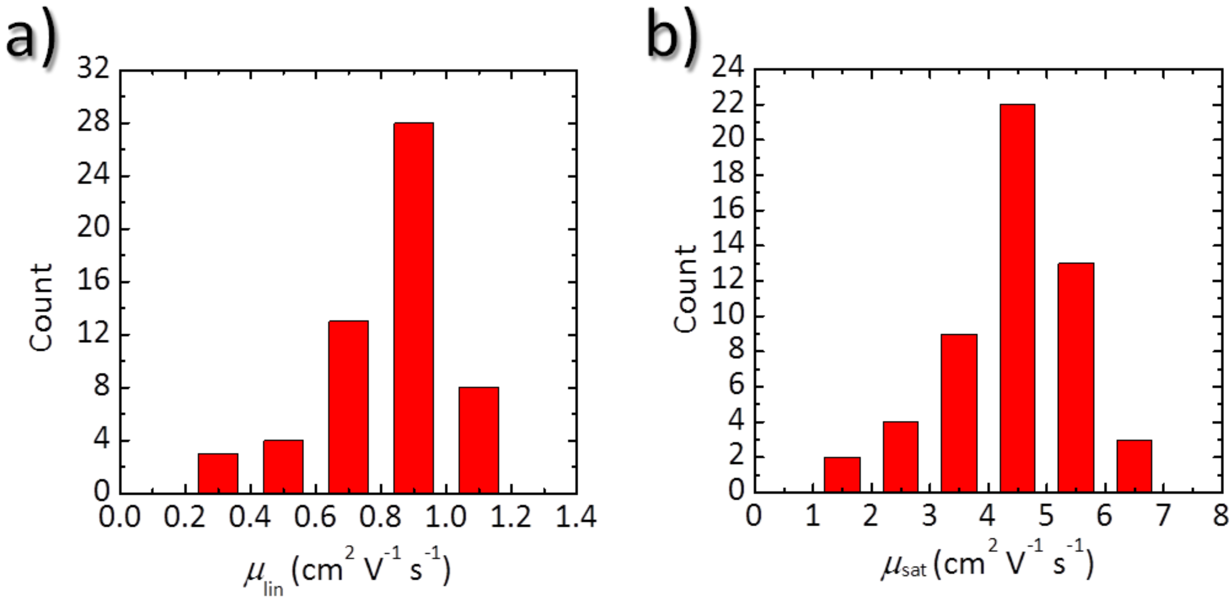

Supplementary Figure 9|Statistics for FETs with transport parallel to coating direction. Statistics for 56 devices FETs with a bar-coated 10 nm thick semiconductor over an area as wide as 8 cm x 8 cm, where charge transport occurs *parallel to the coating direction.* a) Linear regime (V_{GS} = 60 V, V_{DS} = 5 V): maximum mobility is 1.2 $cm^2 V^{-1} s^{-1}$, average mobility is 0.82 $cm^2 V^{-1} s^{-1}$, with a standard deviation of 0.19 $cm^2 V^{-1} s^{-1}$. b) Saturation regime (V_{GS} = 60 V, V_{DS} = 60 V, on the right): maximum mobility is 6.4 $cm^2 V^{-1} s^{-1}$, average mobility is 4.11 $cm^2 V^{-1} s^{-1}$, with a standard deviation of 1.36 $cm^2 V^{-1} s^{-1}$.

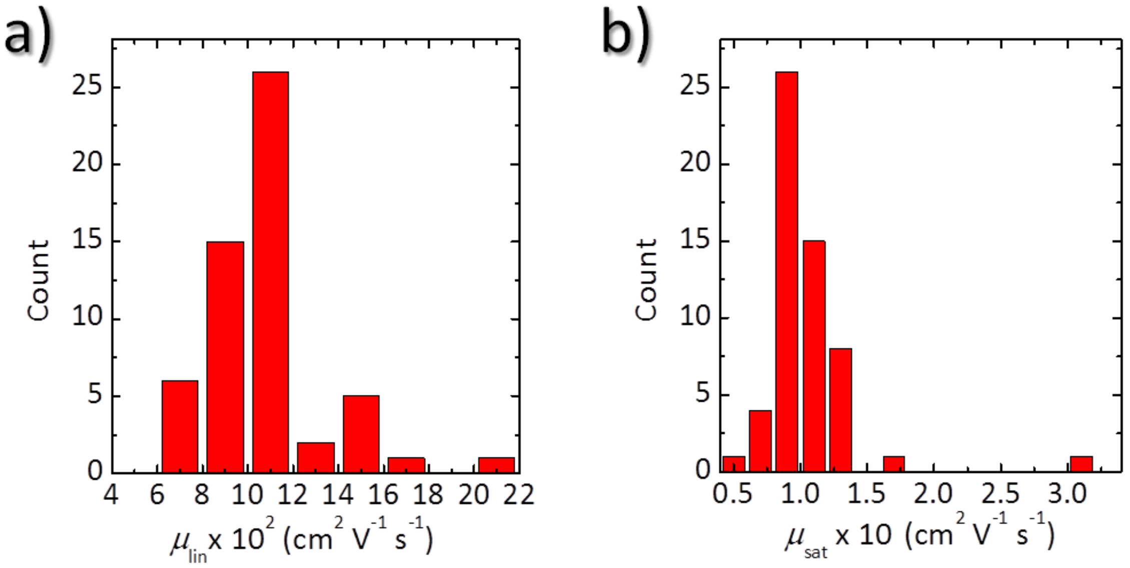

Supplementary Figure 10|Statistics for FETs with transport perpendicular to coating direction. Statistics for 56 devices FETs with a bar-coated 10 nm thick semiconductor over an area as wide as 8 cm x 8 cm, where charge transport occurs *perpendicular to the coating direction.* a) Linear regime (V_{GS} = 60 V, V_{DS} = 5 V): maximum mobility is 0.2 cm^2 V^{-1} s^{-1}, average mobility is 0.1 cm^2 V^{-1} s^{-1}, with a standard deviation of 0.02 cm^2 V^{-1} s^{-1}. b) Saturation regime (V_{GS} = 60 V, V_{DS} = 60 V, on the right): maximum mobility is 0.3 cm^2 V^{-1} s^{-1}, average mobility 0.1 cm^2 V^{-1} s^{-1}, with a standard deviation of 0.03 cm^2 V^{-1} s^{-1}.

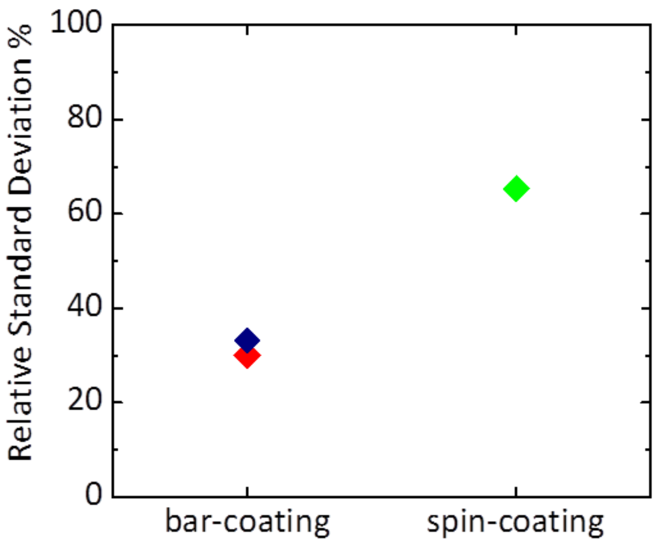

Supplementary Figure 11|Standard deviation of mobility values. Relative standard deviation (*RSD*) values for mobility calculated on the bar-coated semiconductor based devices (red spot, perpendicular *RSD* and blue, parallel *RSD*) and on the spin-coated semiconductor based devices (green spot). The *RSD* has been calculated using the formula: $\boldsymbol{RSD} = \frac{\boldsymbol{S}}{\bar{\boldsymbol{\mu}}}\mathbf{100}$ in which $\boldsymbol{S}$ is the standard deviation and $\bar{\boldsymbol{\mu}}$ the average mobility.

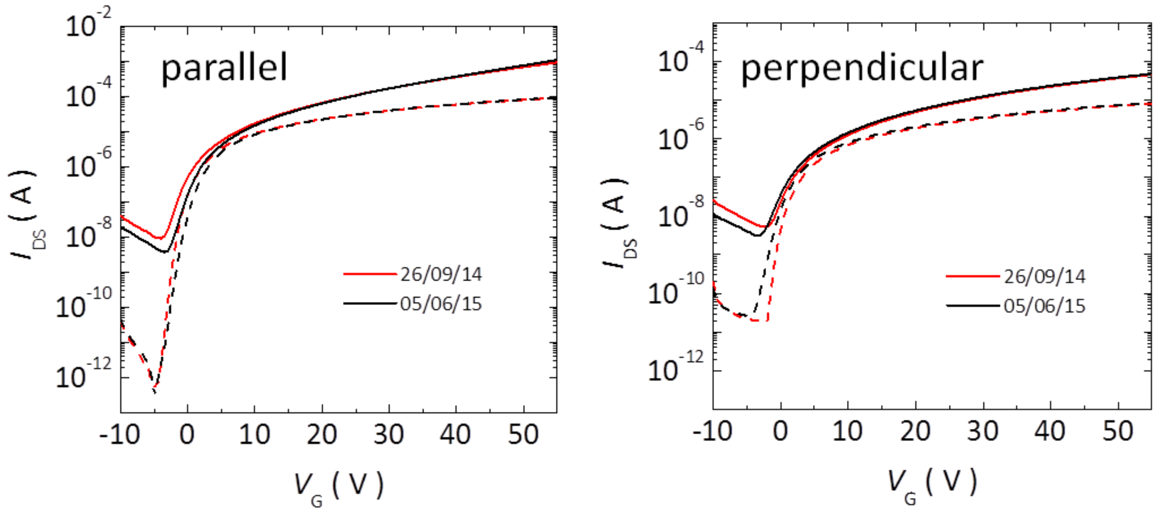

Supplementary Figure 12|FET shelf life stability in inert atmosphere. Transfer curves in linear (V_{DS} = 5 V, dashed lines) and saturation (V_{DS} = 60 V, continuous lines) of freshly fabricated devices (red) and of the same devices measured after 8 months (black) in the parallel (left) and perpendicular (right) cases. We have compared the FET performances after more than 8 months storage at room temperature in nitrogen. We cannot observe any significant degradation, both for the parallel and perpendicular conditions. Shelf life stability in nitrogen is therefore very good and it completely excludes any relevant variation to the morphology sustaining charge transport has occurred.

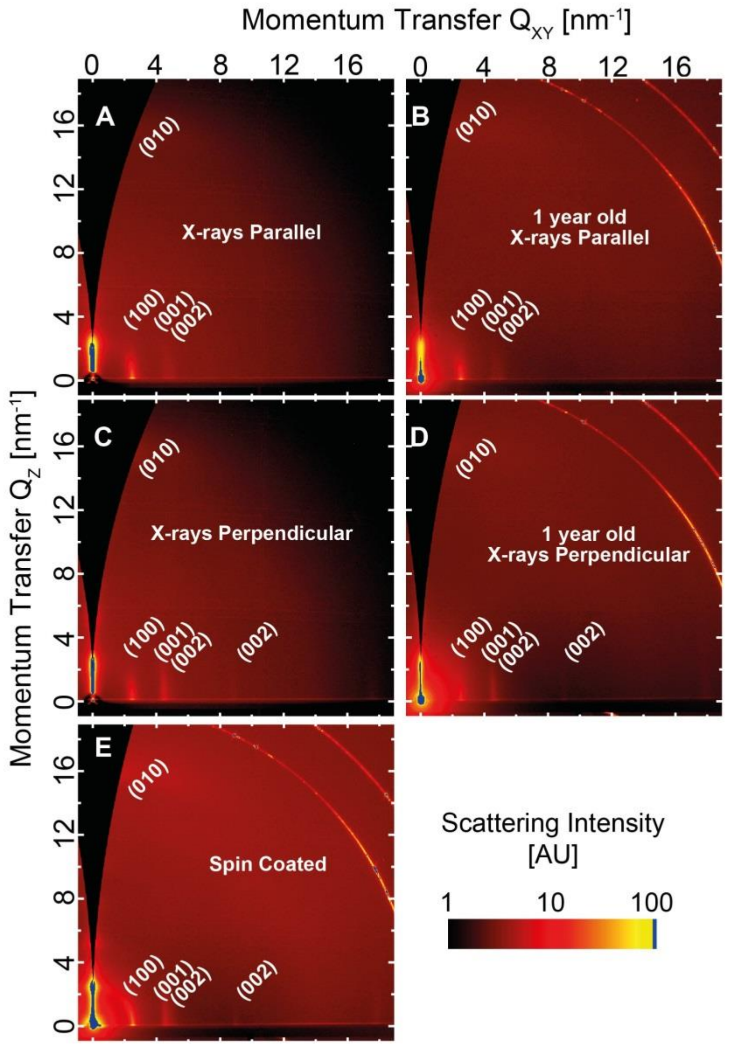

Supplementary Figure 13|GIWAXS measurements on aged and isotropic films. 2D GIWAXS scattering patterns of thin bar-coated films from a 5 g l^{-1} mesitylene recorded with the x-ray beam incident (A,B) parallel and (C,D) perpendicular to the coating direction. A and C were measured on a freshly deposited film, while B and D on a film aged in ambient at room temperature for 1 year. The measurements show that there is negligible morphology evolution in the aged sample. Samples E refers to an isotropic sample deposited by spin-coating from a 5 g l^{-1} mesitylene solution, which have the same crystal packing of bar-coated films.

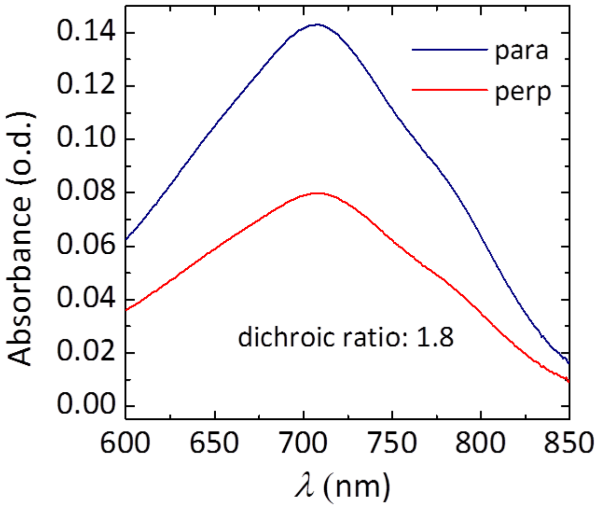

Supplementary Figure 14|Polarized UV-Vis spectra of a bar-coated film. Using this technique, within the bulk of the macro-area probed by the wide polarized spot, an average optical dichroic ratio of 1.8 was measured. This value indicates a limited capability of bar coating to align polymer backbones when compared to other techniques recently applied to the same material.[4] However, the strongly aligned domains observed by AFM appear to contradict the limited dichroic ratio, and suggest a possible discrepancy between surface and bulk alignment. The polarized absorption spectra were measured with a Perkin Elmer lambda 950 spectrophotometer.

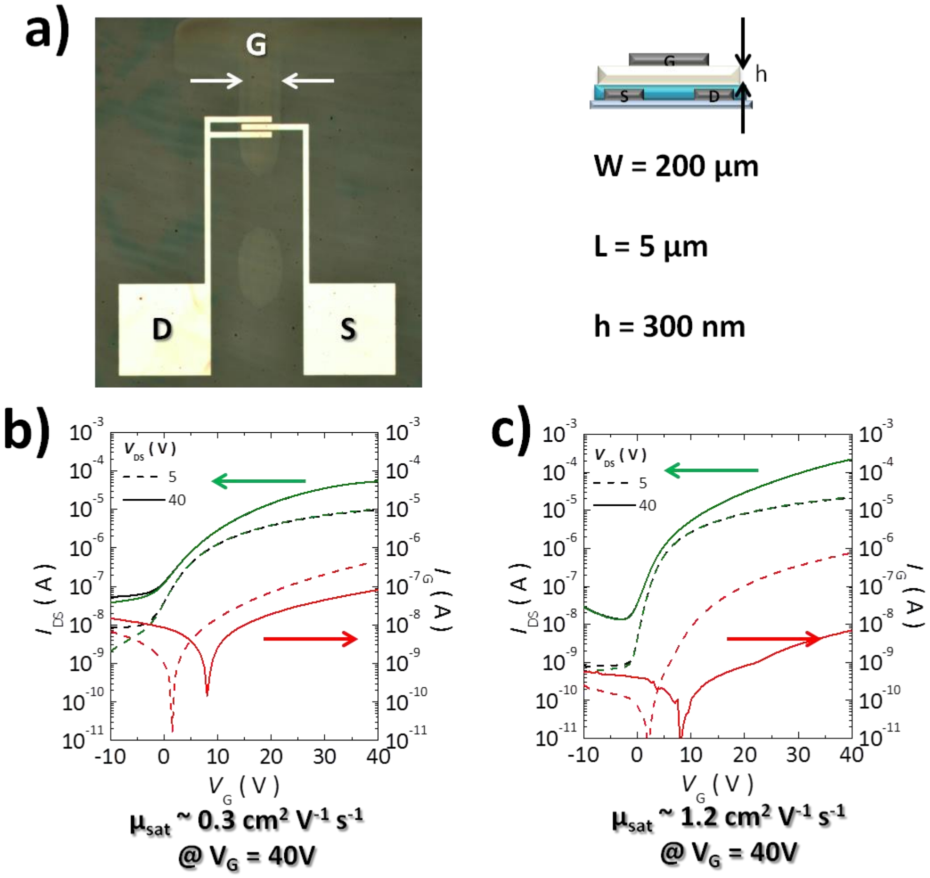

Supplementary Figure 15|FET devices adopted for ac characterization. The devices used for the ac characterization were initially investigated in dc mode by transfer characteristic measurements. a) Optical microscopy picture and a sketch of the side view of a device use for the frequency characterization. The geometry used for the characterization presents a channel width (W) of 200 µm, a channel length (L) of 5 µm and a thickness of the dielectric (d) of 300 nm. The finger width (W_{finger}) is 20 µm. The overlap area between Gate and Source is approximately A_{gs} = 2000 µm^2 while the overlap area between Gate and Drain is approximately A_{gd} = 4000 µm^2. b) Transfer characteristic measured on a device with the semiconductor deposited by spin-coating of a solution in o-DCB. c) Transfer characteristic measured on a device with the semiconductor deposited by bar-coating of a solution in mesitylene.

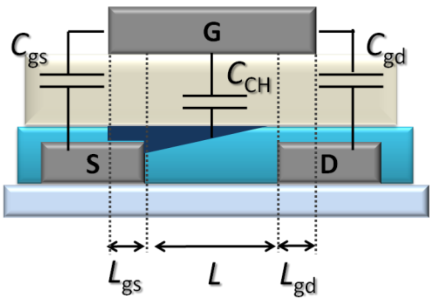

Supplementary Figure 16|Simplistic sketch of the FETs in saturation. L_{gs} and L_{gd} are the overlap length of gate/source and gate/drain and C_{gs}, C_{gd} and C_{CH} are the capacities due to the gate/source, gate/drain and gate/channel overlap. We consider $C_{CH} = C_{CH\text{-}s} + C_{CH\text{-}d}$ as the sum of gate/channel close to source and gate/channel close to drain overlap capacitance.

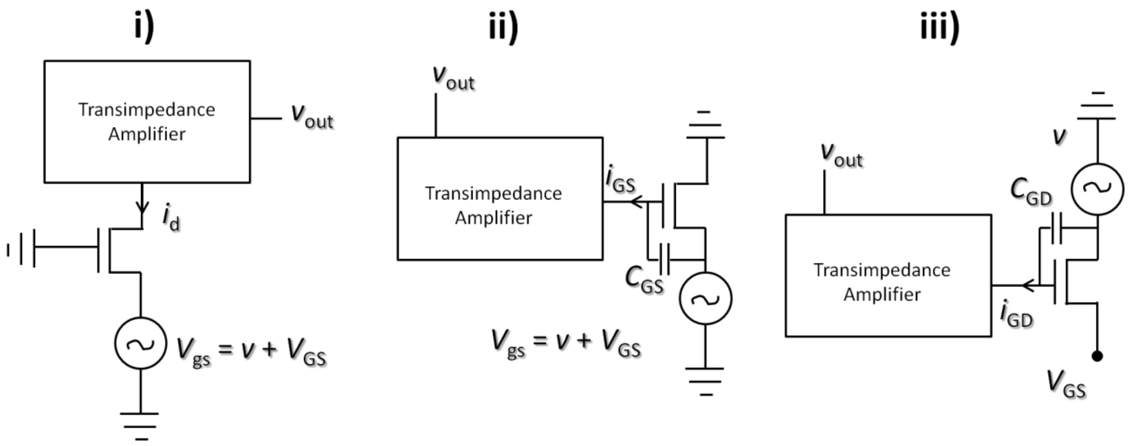

Supplementary Figure 17|Circuit schemes adopted for ac measurements. Scheme of the circuits adopted to extract g_m (i), C_{GS} (ii) and C_{GD} (iii).

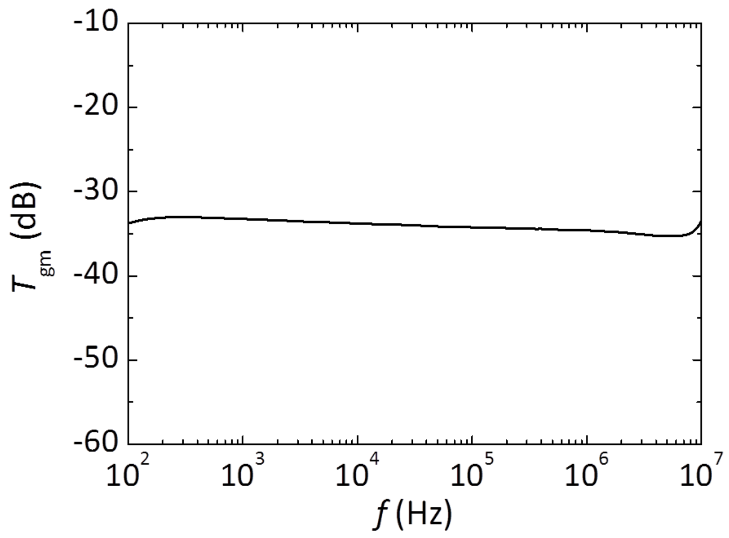

Supplementary Figure 18|FET transconductance. Transfer function measured in the configuration i) of Supplementary Figure 17.

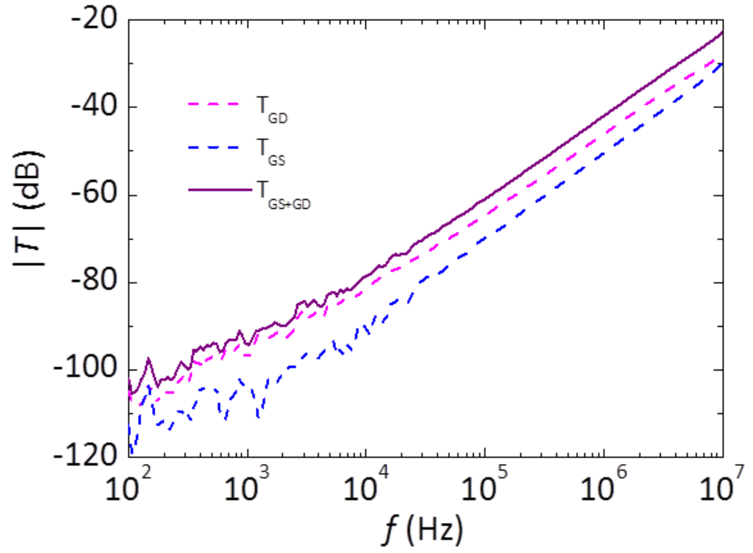

Supplementary Figure 19|Measurement of C_{GS} and C_{GD}. Transfer functions measured in the configuration ii) (blue dashed line), and configuration iii) (pink dashed line) of Supplementary Figure 17, and the sum of the two contribution (purple continuous line).

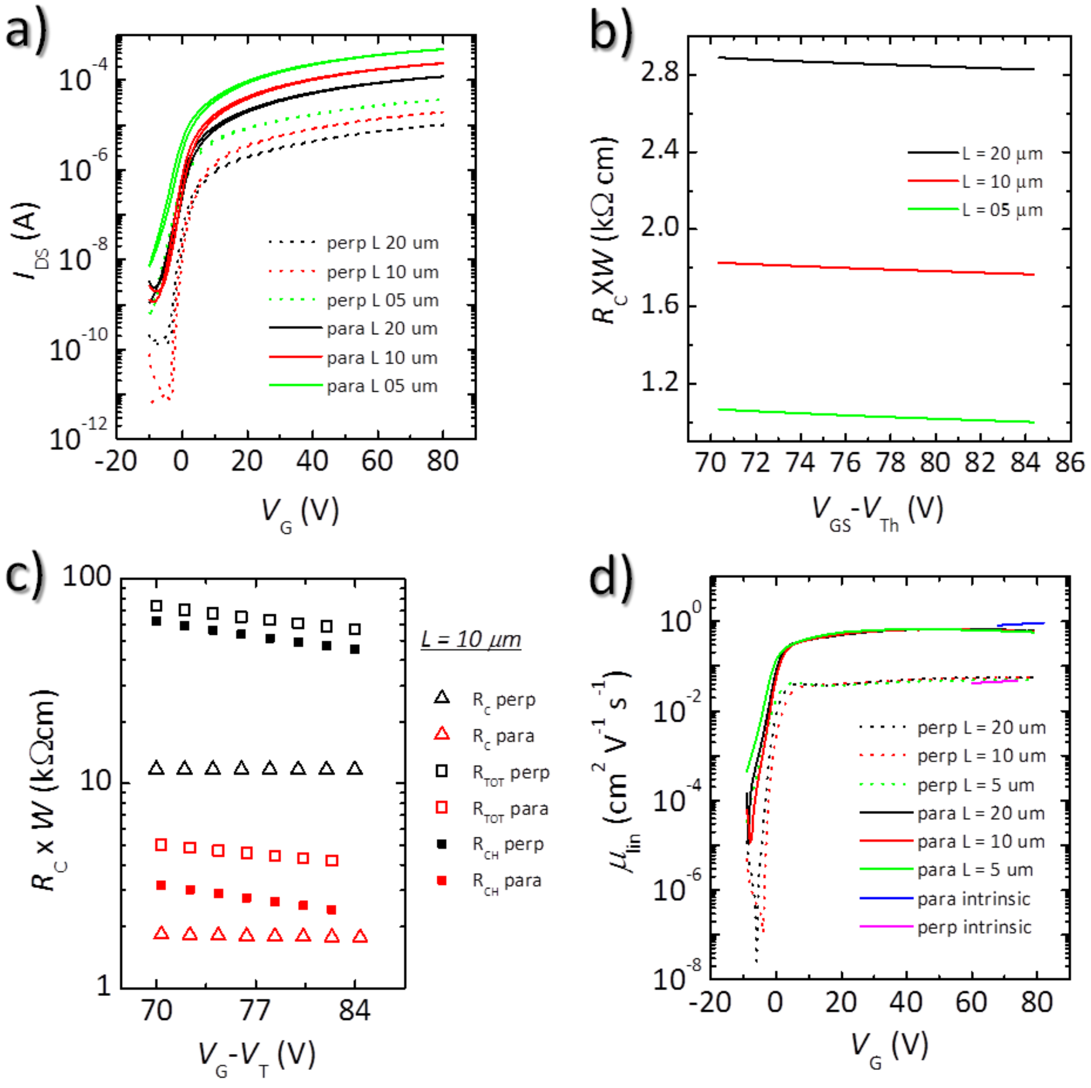

Supplementary Figure 20|Assessment of contact resistance. a) Typical linear transfer curves (V_{DS} = 5 V) of FETs with channel lengths of 20 µm (black), 10 µm (red) and 5 µm (green) channel length, both with the transport probed parallel (solid lines) and perpendicular (dotted lines) to the coating direction. b) Contact resistance versus $V_{GS} – V_{Th}$ for channel length of 20 µm (black), 10 µm (red) and 5 µm (green). c) Normalized contact resistance (triangles), total device resistance (empty squares) and channel resistance (filled squares), in the parallel (red) and perpendicular (black) case, for a device with L = 10 µm. d) Effective linear mobilities for all channel lengths, compared with intrinsic mobilities for the perpendicular (magenta line) and parallel (blue line) case.

Supplementary Tables

Supplementary Table 1. Viscosity data for a 5 g l^{-1} P(NDI2OD-T2) formulation (M_n = 22.2 kDa, PDI = 2.6), measured by Brookfield DV-I Prime viscometer at 25 ^oC.

**Entry**	**Temperature Reading**	**Speed (RPM)**	**Shear Rate (s^{-1})**	**Torque (%)**	**Viscosity (mPa·s)**
1	25.0 ^oC	100	750	52.6	1.61
2	25.0 ^oC	60	450	31.1	1.58
3	25.0 ^oC	50	375	27.1	1.66
4	25.0 ^oC	30	225	16.0	1.62
5	25.1 ^oC	20	150	10.8	1.66
6	25.1 ^oC	10	75	5.4	1.66

Supplementary Table 2. Viscosity data for a 0.5 g l^{-1} P(NDI2OD-T2) formulation (M_n = 22.2 kDa, PDI = 2.6), measured by Brookfield DV-I Prime viscometer at 25 ^oC.

**Entry**	**Temperature Reading**	**Speed (RPM)**	**Shear Rate (s^{-1})**	**Torque (%)**	**Viscosity (mPa·s)**
1	24.9 ^oC	100	750	24.9	0.76
2	24.9 ^oC	60	450	14.9	0.76
3	24.9 ^oC	50	375	12.3	0.75
4	25.0 ^oC	30	225	7.2	0.75
5	25.0 ^oC	20	150	4.8	0.75

Supplementary Table 3. Structural parameters extracted by GIWAXS measurements on samples obtained from a 5 g l^{-1} mesitylene solution deposited by bar-coating and spin-coating. Also reported the parameters extracted from the same bar-coated sample aged for 1 year in ambient atmosphere at room temperature.

	**Alkyl Stacking**					**π-stacking**			**Backbone repeat**			
	**Spacing [nm]**	**Coherence [nm]**	**Dichroic Ratio (para/ perp)**	**Orientation S parameter**	**Relative crystallinity**	**Spacing [nm]**	**Coherence [nm]**	**Relative crystallinity**	**Spacing [nm]**	**Coherence [nm]**	**Dichroic Ration (para/perp)**	**Relative crystallinity**
**Bar coated sample - fresh**	2.52(1)	18(5)	1.27	-0.44	N/A	0.392(1)	3.2(1)	N/A	1.38(1)	20(10)	0.129	N/A
**Spin coated sample - fresh**	2.50(1)	22(1)	N/A	-0.37	1	0.377(1)	3.3(1)	1	1.37(1)	20(5)	N/A	1
**Bar coated sample – aged 1 year**	2.46(1)	18(1)	1.41	-0.45	1.5	0.381(4)	3.1(1)	1.5	1.36(1)	18(5)	0.048	2.2

Supplementary Notes

Supplementary Note 1|Bar-coating process. Bar-coating, also known as rod-coating, is a very well coating technique, part of the “doctoring technique” which comprises also blade-coating, adopting the so called Mayer bars to deposit an ink with a specific wet thickness on a substrate. Mayer bars are typically stainless steel bars which are wound with a wire of a specific diameter. The bar is placed in contact with the substrate and an excess of ink is doctored off as the bar is passed on it. It is the dimension of the gaps between the wound wire around the bar which determines the amount of fluid which can pass through. The fluid then coalesces and levels off to form a uniform wet film as an effect of surface tension. A rough estimation of the wet layer thickness is 10 % of the wound wire diameter. Evaporation of the solvent occurs after the coating, and the final film thickness depends on the solute concentration and its density. The interested reader can refer to [5,6].

We adopted a stainless-steel bar with an application length of 320 mm, purchased from TQC B.V., with a stainless-steel wound wire of 50 μm of diameter (TQC spiral bar applicator), a picture of the adopted bar, with a close up on the wire, is shown in Supplementary Figure 5). We suspended the target substrate on a plastic web of a custom roll-to-roll coater and we deposited 100 μl of solution on the substrate in front of the bar. A wet film was formed by moving the substrate below the bar at a typical coating speed of 3 m min^{-1}. The evaporation of the solvent occurs on a time scale of ~30 s. In the manuscript we show the room-temperature coating of two different polymer solutions in mesitylene, one with a concentration of 5 g l^{-1}, which forms ~10 nm thick films, and one of 0.5 g l^{-1}, which forms submonolayers. We have performed viscosity measurements on both solutions with a Brookfield DV-I Prime viscometer at 25 ^{o}C (spindle CPA-40Z). Results are shown in Supplementary Table 1 and Supplementary Table 2, respectively. Both solutions behave like a Newtonian fluid in the range from 10 to 100 RPM (75 to 750 s^{-1}), with the higher concentration

solution showing a constant viscosity of ~1.6-1.7 mPa·s, and the lower concentration one showing a viscosity of ~0.75-0.76 mPa·s.

Shear rates in bar-coating are typically estimated with a very coarse approximation by taking into account the curved gap between wires.[5,7] In our case, with a wire diameter of 50 μm and a velocity of 3 m min^{-1}, the shear rate is of the order of 2000 s^{-1}. This value lies in the lower range of shear rates developed in roll coatings (100 - 10^5 s^{-1}) or other processes typically adopted to print functional materials, such as slot-die (10^3 – 10^5 s^{-1}).[5,8]

Under these conditions, we can estimate the Reynolds number $Re = \rho v l \mu^{-1}$ (ρ density of the ink, v velocity, l characteristic length scale which for rod coating is the diameter of the wire, μ viscosity) adopting the constant viscosity of both formulations in the available shear rate range. We obtain $Re \approx 1.0$ for the 5 g l^{-1} solution and $Re \approx 2.3$ for the 0.5 g l^{-1}. These numbers indicate that we are far below the onset of turbulence, thus ensuring a laminar flow. Although we have not measured the viscosity exactly up to 2000 s^{-1}, the Newtonian behavior of the fluids in the investigated range, with no evident deviation, makes the estimation of *Re* sufficiently robust.

Supplementary Note 2|Quantification of contact resistance. In order to quantitavely characterize contact resistance R_C in the devices reported in this work, we have adopted the perpendicular case by adopting the "differential method" (DM)[9] in the linear regime on a series of devices on devices with channel lengths of 5, 10 and 20 μm, comprising at least 4 samples per each channel.

In Supplementary Figure 20a the typical transfer curves in the linear regime (V_{DS} = 5 V) for devices with different channel lengths, both in the parallel and perpendicular direction, are reported. The strong anisotropy is present in all devices. Importantly, already at a qualitative level, the currents do scale almost linearly with $1/L$, both in the parallel and perpendicular directions. This is a first indication of a reduced effect of the contact resistance.

The DM fits best in the high gate voltage range, between 70 and 85 V; therefore this range has been used to reliably extract the contact resistance. The result of the analysis is shown in the Supplementary Figure 20b for all channel lengths. In the case for example of 10 μm long channel devices (Supplementary Figure 20c), the contact resistance for the parallel case is ~ 1.8 kΩ cm, while it is ~ 12 kΩ cm for the perpendicular case. A difference in contact resistance is expectable since it is very well known that in organic semiconductors charge injection efficiency depends on charge mobility.[10] For a device with 10 μm long channel, in the perpendicular case the contact contributes only 16 % of the total device resistance (R_{TOT}), the 84 % percent being represented by the channel resistance (R_{CH}, Supplementary Figure 20c). In the parallel case the contact contribution percentage is a bit higher, being ~35 %.

To better visualize the effect of the contact resistance on mobility extraction, a comparison between effective and linear mobility is proposed in Supplementary Figure 20d. In fact, by quantitatively knowing the contact resistance, it is possible to extract the intrinsic linear mobility, therefore the mobility of the devices depurated by the contact effect. We have plotted the linear mobilities for all channels and directions in Supplementary Figure 20d, along with the intrinsic mobility reliably extracted in the high gate voltage range. The effective mobilities for all channel lengths are basically the same, they all show a strong anisotropy and they are very close to the intrinsic mobility values, as an effect of the limited contact resistance.

Supplementary Methods

Supplementary Method 1|Assessment of the transition frequency. The transition frequency f_t indicates the upper limit for the operative frequency of a transistor and represents an important figure of merit for the evaluation of the device performance in a real application. A detailed investigation of this parameter is important in the evaluation of a FET ac operation. To extract f_t, we start from Caironi's work.[11] By modulating the gate/source voltage with a small ac signal v_{gs} around a bias point V_{GS}, the current flowing in the channel is the sum of a static component I_D and an ac component i_d, proportional to v_{gs}:

$$i_{\mathrm{d}} = g_{\mathrm{m}} v_{\mathrm{gs}}$$

in which g_m is the transconductance of the device and it is proportional to the mobility μ and to L^{-1}.

However, during the frequency characterization of an FETs in top gate, bottom staggered electrodes, in which the gate electrode overlap both the source and drain contacts, as reported in Supplementary Figure 16, the i_d depends on g_m as well as on the paracitic overlap capacities and channel capacity.

We can then introduce the following gate capacitive current:

$$i_{\mathrm{g}} = i_{\mathrm{GS}} + i_{\mathrm{GD}} = j2\pi f(C_{\mathrm{GS}} + C_{\mathrm{GD}})v_{\mathrm{gs}}$$

in which $C_{\mathrm{GS}} = C_{\mathrm{gs}} + C_{\mathrm{CH-s}}$ and $C_{\mathrm{GD}} = C_{\mathrm{gd}} + C_{\mathrm{CH-d}}$. The total current that flows from source to drain finally is:

$$i_{\mathrm{D}} = i_{\mathrm{d}} - i_{\mathrm{g}}$$

At this point we can define the transition frequency f_t as the frequency where $i_d = i_g$: below this frequency the i_d current dominates and i_D is constant in frequency (ideal behaviour), and above it current increases with frequency as it is dominated by i_g.

$$f_{\mathrm{t}} = \frac{g_{\mathrm{m}}}{2\pi(C_{\mathrm{GS}} + C_{\mathrm{GD}})}$$

For the extraction of the three parameters that define f_{t} we used the schematic configuration represented in the sketch in Supplementary Figure 17. Firstly we measured the ac component, excluding the capacitive currents, which depends only on the transit time of the carriers (i) then we measured the capacitive contribution due to C_{GS} (ii) and C_{GD} (iii) and we calculated the fully gate capacitance $C_{\mathrm{G}} = C_{\mathrm{GS}} + C_{\mathrm{GD}}$, summing the two contributions. We used an Agilent ENA series network analyzer to modulate the ac voltage signal, from 1 Hz to 10^8 Hz, and a transimpedance amplifier, with a gain G, that returned an ac voltage v_{out} to the network analyzer, to read the ac currents produced. The system finally displayed the transfer function $v_{\mathrm{out}}\, v^{-1}$. The devices were characterized in saturation regime ($V_{\mathrm{G}} = V_{\mathrm{D}}$).

In order to determine g_{m}, the source electrode was modulated with a signal v around a fixed bias V_{GS} = -40V and the transimpedance amplifier was connected to the drain electrode. The transfer function measured by the network analyzer on a device with the semiconductor deposited by bar coating from mesitylene is reported in Supplementary Figure 18.

In this situation we extracted g_{m} as follows:

$$i_{\mathrm{d}} = -v g_{\mathrm{m}}$$

The current i_d was read and amplified by the transimpedance amplifier giving:

$$v_{\mathrm{out}} = -i_{\mathrm{d}} G$$

So:

$$\frac{v_{\mathrm{out}}}{v} = G g_{\mathrm{m}} = T_{\mathrm{gm}}[dB]$$

Knowing the gain of the amplifier ($G = 2 \times 10^3$), g_{m} is:

$$g_{\mathrm{m}} = \frac{10^{\frac{T_{\mathrm{gm}}}{20}}}{G}$$

Here are reported the values calculated on a device made by spin-coating the semiconductor from o-DCB and on a device made by bar-coating of the semiconductor from mesitylene.

- $g_{\mathrm{m-oDCB}} = 2\frac{\mu A}{V}$
- $g_{\mathrm{m-mesitylene}} = 14\ \frac{\mu A}{V}$

In order to determine C_{GS} and C_{GD}, as the case i), the source electrode was modulated with a signal v around a fixed bias V_{GS} = -30V and the transimpedance was connected to the gate electrode, reading the capacitive current that flows through the gate/source overlap. The same was done for the measurement of the capacitve current that flows through the gate/drain overlap, but differently, the source electrode was fixed at a bias V_{S} = -30V, the drain was modulated with a signal v around zero voltage and the transimpedance was connected to the gate electrode. In Supplementary Figure 19 the transfer functions related to the capacitive current between gate and source (blue dashed line), gate and drain (pink dashed line) and the sum (purple continuous line) are represented.

At high frequency (f > 10^5 Hz), the slope of the transfer functions is 20dB dec^{-1}, that of a capacitive current.

We extract the $C_{\mathrm{G}} = C_{\mathrm{GS}} + C_{\mathrm{GD}}$ as follows:

$$i_{\mathrm{G}} = i_{\mathrm{GS}} + i_{\mathrm{GD}} = -v2\pi f(C_{\mathrm{GS}} + C_{\mathrm{GS}})$$

The current i_{GS} and i_{GD} were read and amplified by the transimpedance amplifier giving:

$$v_{\mathrm{out}} = -i_{\mathrm{G}}G$$

So:

$$\frac{v_{\mathrm{out}}}{v} = 20\log(f) + 20\log(2\pi f(C_{\mathrm{G}})\mathrm{G} = T_{\mathrm{GS+GD}}[\mathrm{dB}]$$

Then for the extraction of C_G, knowing the gain G:

$$C_{\mathrm{G}} = \frac{10^{\frac{T_{\mathrm{GS+GD}}}{20}}}{2\pi G f}$$

Here are reported the values calculated on a device made by spin-coating the semiconductor from o-DCB and on a device made by bar-coating of the semiconductor from mesitylene, extracted at f = 1 MHz.

- $C_{\mathrm{G-oDCB}} = 0.712\ pF$
- $C_{\mathrm{G-mesitylene}} = 0.596\ pF$

Supplementary References

1 Luzio, A. *et al*. Control of charge transport in a semiconducting copolymer by solvent-induced long-range order. *Sci. Rep.* **3**, 3425 (2013).

2 Matsidik, R. *et al*. Defect-free Naphthalene Diimide Bithiophene Copolymers with Controlled Molar Mass and High Performance via Direct Arylation Polycondensation. *J. Am. Chem. Soc.* **137**, 6705-6711 (2015).

3 Steyrleuthner, R. *et al.* Aggregation in a high-mobility n-type low-bandgap copolymer with implications on semicrystalline morphology. *J. Am. Chem. Soc.* **134**, 18303-18317 (2012).

4 Tremel, K. *et al.* Charge Transport Anisotropy in Highly Oriented Thin Films of the Acceptor Polymer P(NDI2OD-T2). *Adv. Energy Mater.* **4**, 1301659 (2014).

5 Göring, W. Modern Coating and Drying Technology. *Materials and Corrosion* **44** (1993).

6 Tracton, A. A. Coatings Technology Handbook. 3rd Ed. CRC Press (2006).

7 Guo, F. *et al.* Vertically aligned graphene layer arrays from chromonic liquid crystal precursors. *Adv. Mater.* **23**, 508-513 (2011).

8 Gutoff, E. B. & Cohen E. D. Coating and drying defects. *Drying Technology* **13**, 2251-2252 (1995).

9 Natali, D. *et al.* M. Modeling of organic thin film transistors: Effect of contact resistances. *J. App. Phys.* **101**, 014501 (2007).

10 Natali, D. & Caironi, M. Charge Injection in Solution-Processed Organic Field-Effect Transistors: Physics, Models and Characterization Methods. *Adv. Mater.* **24**, 1357-1387 (2012).

11 Caironi, M., Noh, Y.-Y. & Sirringhaus, H. Frequency operation of low-voltage, solution-processed organic field-effect transistors. *Semicond. Sci. Technol.* **26**, 034006 (2011).
